# A new tool for engineering *Phaeodactylum tricornutum*: the *METE* promoter drives both high expression and B_12_ ‐tuneable regulation of transgenes

**DOI:** 10.1111/tpj.70210

**Published:** 2025-10-21

**Authors:** Patrick R. Hickland, Katrin Geisler, Shelby Newsad, Marcel Llavero Pasquina, A. Caroline Faessler, Gonzalo I. Mendoza‐Ochoa, Andre Holzer, Payam Mehrshahi, Alison G. Smith

**Affiliations:** ^1^ Department of Plant Sciences University of Cambridge Downing Street Cambridge CB2 3EA UK; ^2^ Present address: Environmental Science and Technology Institute (ICTA‐UAB) Universitat Autònoma de Barcelona (UAB) Bellaterra (Cerdanyola del Vallès) 08193 Spain; ^3^ Present address: MRC Laboratory of Molecular Biology Francis Crick Avenue, Cambridge Biomedical Campus Cambridge CB2 0QH UK; ^4^ Present address: Holzer Scientific Consulting GmbH Campus Starterzentrum Saarbrücken 66123 Germany

**Keywords:** microalgae, *Phaeodactylum tricornutum*, tuneable promoter, vitamin B_12_, metabolic engineering, diterpene, methionine synthase, enhancer motif

## Abstract

For advanced metabolic engineering strategies, it is crucial to be able to regulate transgene expression, to prevent potential deleterious effects in the host organism during growth and allow optimisation of production levels. Here, we identified vitamin B_12_ (cobalamin)‐responsive promoters in the diatom *Phaeodactylum tricornutum,* a promising biotechnological chassis that readily absorbs this metabolite with minimal physiological impact. Using promoter–reporter constructs, the promoters of the cobalamin acquisition protein 1 (CBA1) and the B_12_‐independent form of methionine synthase (METE) were shown to regulate transgene expression in a B_12_‐dependent manner. Further characterisation of the *METE* promoter (*P*_*METE*_) demonstrated that it exhibited significantly higher expression levels than several previously characterised promoters, but could be repressed by nanomolar amounts of B_12_, with a dynamic range >100‐fold. Tight regulation was demonstrated by the suppression of the lethal ribonuclease, barnase at 1 μg L^−1^ B_12_. Reporter expression was doubled when P_*METE*_ was paired with its cognate terminator, compared with the widely used *FCPA* terminator. Promoter truncations resulted in decreased expression, but no loss of B_12_ regulation. A 14 nucleotide motif, present in four copies in P_*METE*_, was found to be necessary for expression, and when fused to the constitutive *FCPA* promoter, enhanced expression levels. Transgenic lines expressing the heterologous diterpenoid enzyme, casbene synthase, produced casbene titres of approximately 2 mg L^−1^ and this was tuneable by B_12_. This demonstrates the utility of P_*METE*_ in efforts to establish *P. tricornutum* as an industrial biotechnology production platform.

## Significance Statement

The promoter of the METE gene in the diatom *Phaeodactylum tricornutum* is repressed by nanomolar concentrations of vitamin B_12_. It can be used to tune the expression of genes for reporter proteins or metabolic enzymes and in the non‐permissive state (i.e., +B_12_) can suppress the expression of the lethal gene, barnase.

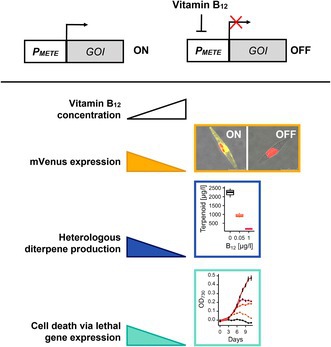

To expand the molecular and synthetic biology toolkit in the marine microalga *Phaeodactylum tricornutum,* we have identified and characterised an endogenous promoter that drives high‐level expression and can be finely regulated by the addition of a benign and inexpensive metabolite. This will facilitate future gene discovery and metabolic engineering efforts in this organism.

## INTRODUCTION

Diatoms are globally distributed unicellular algae responsible for up to 20% of global primary productivity (Field et al., 1998; Gregg and Rousseaux, 2014). Among them, *Phaeodactylum tricornutum* has attracted considerable attention from the biotechnology industry for its productivity and the range of valuable products that can be derived from its biomass. As with most algal species of note, the high levels of lipid accumulation exhibited by *P. tricornutum* have resulted in interest in its use as a biofuel feedstock. However, *P. tricornutum* also produces the omega‐3 fatty acids eicosapentaenoic acid and docosahexaenoic acid as well as the carotenoid fucoxanthin, a commercially important nutraceutical for both the human and aquaculture markets (Kim et al., 2012).

As well as its industrial relevance, *P. tricornutum* has one of the most developed sets of resources for genetic manipulation amongst microalgae (Falciatore et al., 2020), with both nuclear (Bowler et al., 2008) and chloroplast (Li et al., 2020) genome sequences available, transcriptomic datasets (Alipanah et al., 2018; Allen et al., 2008; Ashworth et al., 2016; Bertrand et al., 2012; Rastogi et al., 2018) and numerous examples of CRISPR editing approaches (Nymark et al., 2016; Serif et al., 2018; Sharma et al., 2021) As a result, it has been used extensively as a chassis for metabolic engineering (D'Adamo et al., 2019; Fabris et al., 2020). However, until recently, the promoters available to drive transgene expression in *P. tricornutum* were limited. The promoters for the light‐harvesting chlorophyll‐fucoxanthin‐binding proteins (*FCP*; also referred to as *LHCF*) (Apt et al., 1996; Falciatore et al., 1999) have been most commonly used (D'Adamo et al., 2019; Hamilton et al., 2016; Radakovits et al., 2011) due to their relatively constant expression during active cell growth. Nonetheless, the activity of these promoters is reduced in the dark (Nymark et al., 2013), in the stationary growth phase (Erdene‐Ochir et al., 2016) as well as during nutrient deprivation, such as nitrogen (Alipanah et al., 2015) or iron (Allen et al., 2008).

In recent years, the molecular toolbox of *P. tricornutum* has expanded with increasing numbers of characterised promoters, such as from the gene for elongation‐factor 2 (P_*EF2*_; Seo et al., 2015) P_*GLNA*_ from the gene for glutamine synthase (Erdene‐Ochir et al., 2016), and P_HASP1_ from the identified highly abundant secreted protein 1 (Erdene‐Ochir et al., 2019), all of which provide stable expression under most growth and nutrient conditions. Ideally, for both fundamental research and biotechnological settings, it is also crucial to have promoters whose expression can be modulated in a controlled way (Brophy and Voigt, 2014). Such regulatable promoters are useful, for example, when gene knock‐outs result in a lethal phenotype. In biotechnology scenarios, engineering microorganisms to produce heterologous compounds, particularly when expressing complex, multi‐step heterologous pathways (Dahl et al., 2013; Feist and Palsson, 2010), can drive competition for substrates and cofactors as well as interference with native cellular processes (Borkowski et al., 2016). This in turn can lead to reduced growth rates and an overall decrease in the productivity of the bioprocess (Wu et al., 2016). As such, the ability to tune the expression of particular genes can ensure sufficient downstream substrate availability, while minimising the accumulation of intermediates that may be toxic. Further, using regulatable promoters can decouple biomass generation from the production of the engineered compound (Sproles et al., 2021).

Regulatable promoters identified in *P. tricornutum* include those responsive to CO_2_ (Harada et al., 2005), iron (Yoshinaga et al., 2014), nitrogen (Chu et al., 2016; Shemesh et al., 2016), and phosphate (Lin et al., 2017). However, using endogenous promoters to underpin inducible systems can have drawbacks. These include the potential for leaky expression and the unintended consequences that may result from exposing cells to potential stress‐inducing conditions. For example, the nitrate reductase gene, *NR*, is nitrate inducible and NH_4_^+^ repressible, but growth is lower under permissible conditions. Moreover, the promoter is leaky, such that a background level of expression is maintained even in the presence of NH_4_^+^ (Poulsen and Kröger, 2005). The P_*AMT*_ from the ammonium transporter confers increased expression throughout nitrogen starvation (Adler‐Agnon et al., 2018), but this reduces growth severely. Similarly, protein expression controlled by the P_*AP1*_ promoter is induced some eightfold under phosphate limitation conditions (Lin et al., 2017), but this also impacts growth and there are extensive changes in other genes, with differential expression of approximately 65% of the genome (Cruz de Carvalho et al., 2016). An alternative approach to using endogenous promoters took advantage of chimeric synthetic systems developed in other chassis. Introduction of the XVE/OlexA promoter responsive to the addition of β‐oestradiol (Zuo et al., 2000) or the DIG/pUAS promoter controlled by digoxin (Feng et al., 2015) into *P. tricornutum*, followed by the addition of the respective ligands, induced reporter gene expression in a dose‐ and time‐dependent manner (Kassaw et al., 2022).

We wanted to develop a tightly regulated expression system for *P. tricornutum* using native sequences that did not suffer from large systems‐level impact in induction conditions. We chose promoters regulated by vitamin B_12_ (cobalamin), which is used as a cofactor for the B_12_‐dependent methionine synthase (METH). No eukaryotic algae are capable of B_12_ biosynthesis, which is produced by a subset of bacteria only (Shelton et al., 2019; Warren et al., 2002), so those that encode METH take up B_12_ from the environment (Croft et al., 2005; Tang et al., 2010). However, for many algae, including *P. tricornutum*, this is not essential because they possess another form of methionine synthase, METE, that does not need B_12_ as a cofactor. As a consequence, in *P. tricornutum* cells grown with or without B_12_ just 6% of transcripts were changed (Bertrand et al., 2012), with minor impacts on the growth rate of cultures, compared with transcript changes of 16% for iron limitation (Allen et al., 2008; Bertrand et al., 2012) or >50% for nitrogen (Alipanah et al., 2015; Levitan et al., 2015) and for phosphate (Cruz de Carvalho et al., 2016) (Table S1). Inspection of the RNAseq dataset from Bertrand et al. (2012) allowed us to identify potential B_12_‐regulatable promoters, which were tested using reporter assays. The *METE* promoter was the most promising and therefore was further characterised to demonstrate effective regulation of different transgenes, including for the production of the commercially relevant diterpene, casbene.

## RESULTS

### Identification of nutrient dependent promoters

**Figure 1:**
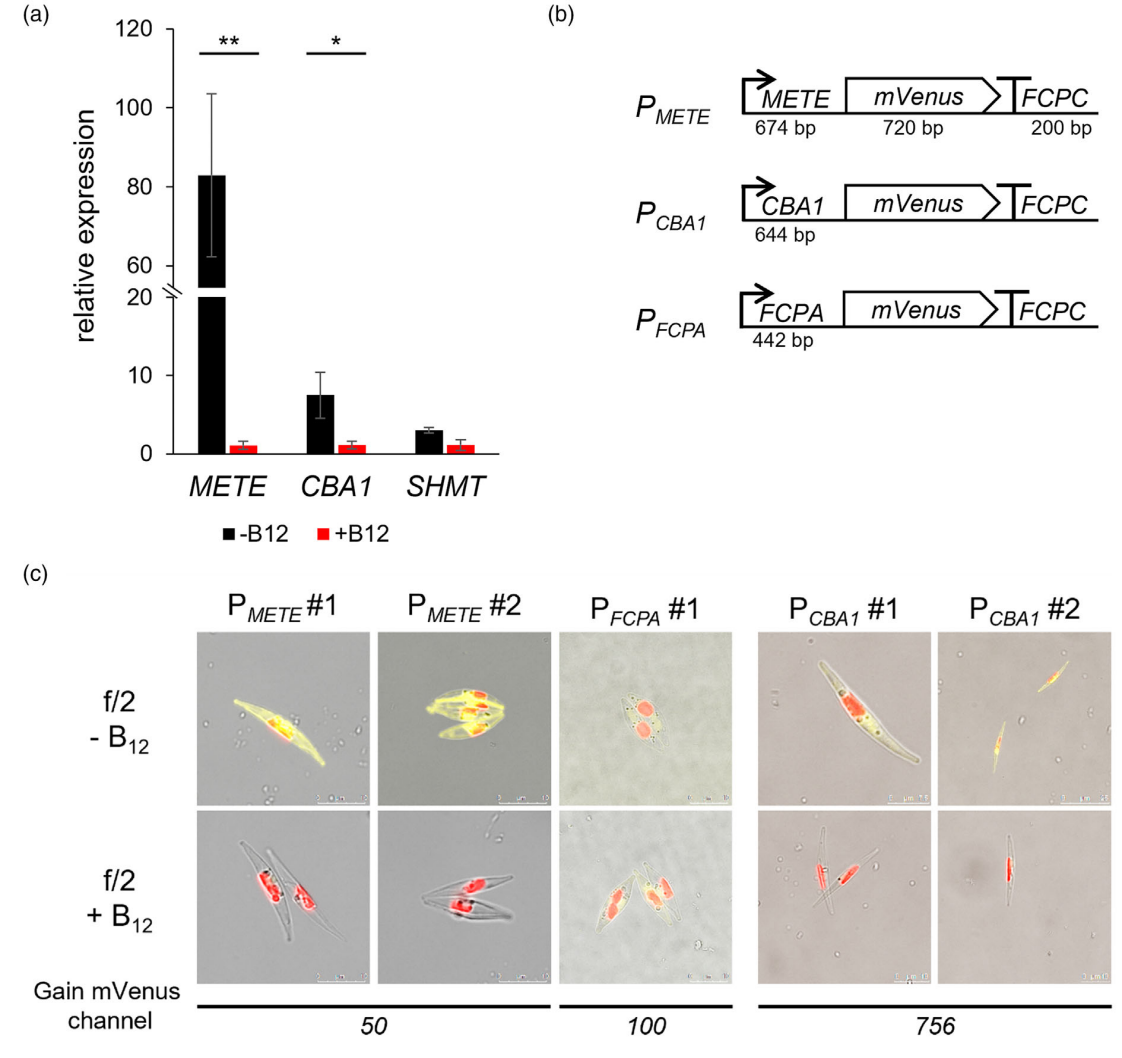
Identification of B_12_ tuneable promoters in *P. tricornutum*. (a) *P. tricornutum* cells were grown in f/2 without supplementation (−B_12_, black bars) or with 1 μg L^−1^ cyanocobalamin (+B_12_, red bars). After 24 h, RT‐qPCR was carried out with primers for the following genes: B_12_‐independent isoform methionine synthase (*METE*), cobalamin acquisition protein 1 (*CBA1*) and serine hydroxymethyltransferase (*SHMT*). The 2^(ΔΔCq)^ values, normalised to the housekeeping gene ubiquitin conjugating enzyme (*UBC*), are shown for each gene. Statistics were analysed with Welch's *t*‐test with *P*‐values shown above boxplots: **P < 0.005, *P < 0.05, *n* = 4. (b) Schematic representation of promoter–reporter constructs. The promoters of *P. tricornutum METE* and *CBA1* genes, together with *FCPA*, were assembled with a mVenus reporter and *FCPC* terminator. Each expression cassette, together with a zeocin resistance cassette, was transformed into *P. tricornutum*. The length of each part is shown in base pairs (bp) under the schematic. (c) Confocal microscope images of transformed *P. tricornutum cells*. Shown is the overlay of separate images of mVenus fluorescence, chlorophyll fluorescence, and brightfield of representative transformants of *METE* promoter lines (P_*METE*_), P_*CBA1*_, and P_*FCPA*_. Cells were grown without or with B_12_ (1 μg L^−1^) and imaged after 4 days. To detect mVenus fluorescence, the gain was adjusted for the different lines, with the values shown underneath. Images were taken with excitation at 515 nm and emission for mVenus at 535–565 nm and emission for chlorophyll at 650–720 nm.

To identify an endogenous promoter for *P. tricornutum* that could be tightly regulated without causing significant physiological changes, we queried several available RNAseq datasets in which the effect of reducing or omitting nutrients was assessed (Table S1). Growth with and without vitamin B_12_ (cobalamin; Bertrand et al., 2012) showed the fewest changes to the overall transcriptome, with three genes identified to be consistently and significantly upregulated in the absence of B_12_, namely, the B_12_‐independent methionine synthase (*METE*), serine hydroxymethyltransferase 1 (*SHMT1*, Phatr3_J18665), and the cobalamin acquisition protein 1 gene (*CBA1*). These have all been shown to respond similarly in the unrelated green alga *Chlamydomonas reinhardtii* (respectively Croft et al., 2005; Helliwell et al., 2014; Sayer *et al*., 2024). To confirm the apparent upregulation in RNAseq analysis, we conducted an RT‐qPCR experiment (Figure 1a). When *P. tricornutum* cells were grown in f/2 media with 1 μg L^−1^ B_12_, transcript levels for *METE* were reduced ~80‐fold after 24 h compared with cells grown without the vitamin. *CBA1* transcripts were some ten times lower than those for METE but were also repressed (~7‐fold) by B_12_. For *SHMT1*, a threefold difference in transcript levels was observed between +/− B_12_ conditions, but this was not significant and, as overall transcript levels of this gene were very low, we continued the analysis with the promoters of *METE* and *CBA1*.

The *METE* gene (*Phatr3_J28056*) encodes a vitamin B_12_‐independent methionine synthase. The coding sequence is 2373 bp in length and contains one intron (Figure S1). In the freshwater alga *C. reinhardtii*, *METE* expression is regulated at the transcriptional level by its promoter (Helliwell et al., 2014). Therefore, we hypothesised that the genetic element responsible for the B_12_‐responsiveness of *P. tricornutum METE* would also be the promoter. To investigate this, we used information from the Ensembl Protist Genome browser (https://protists.ensembl.org/index.html) and the *P. tricornutum* genome assembly v3 to identify the upstream sequence. A 674 bp region starting −1 bp from the ATG start codon up to the coding sequence of the neighboring gene, *Phatr3_J46628*, was taken to encompass the promoter and 5′ untranslated region (5′UTR), hereafter referred to as P_*METE*_. Primers used to amplify the fragment from *P. tricornutum* genomic DNA are listed in Table S2. The protein encoded by the *CBA1* gene (*Phatr3_J48322*) has been shown to be involved in B_12_ uptake in *P. tricornutum* by studying CRISPR/Cas9 knockout mutants (Sayer et al., 2024). As for *METE*, we identified the *CBA1* promoter/5′UTR region as corresponding to the 644 bp from the end of the upstream gene (*Phatr3_J29488*) to −1 bp from the start codon (Figure S1) and amplified from genomic DNA. Both P_METE_ and P_CBA1_ fragments were cloned upstream of the gene encoding the yellow fluorescent protein, *mVenus* (Figure 1b), as was the routinely utilised constitutive promoter P_*FCPA*_ (Apt et al., 1996). The *FCPC* terminator (T_*FCPC*_) was used as a terminator in all reporter constructs analysed in this initial experiment and the *Ble* gene for zeocin‐resistance was included as a selectable marker. Hereafter, these promoter‐mVenus‐T_*FCPC*_ constructs are referred to simply by the promoter used.

The reporter constructs were introduced into wild‐type *P. tricornutum* CC1055‐1 cells by electroporation, and transformants were selected on f/2 agar plates containing 75 mg L^−1^ zeocin. Because of the random integration of transgenes to mitigate position effects, multiple transgenic lines showing reporter gene expression were identified for each construct and maintained in liquid f/2 + zeocin. Transformed lines were imaged by confocal microscopy after culturing in media with and without 1 μg L^−1^ B_12_ (Figure 1c, Figure S2). Images of P_*METE*_ lines showed strong mVenus fluorescence in cells grown without B_12_, but no fluorescence was detected when B_12_ was present. In contrast, the mVenus signal in the P_*FCPA*_ line did not change. For P_*CBA1*_ lines, there was lower fluorescence overall in the absence of the vitamin, requiring the gain on the microscope to be increased considerably to detect fluorescence; nonetheless, addition of B_12_ still suppressed expression. Although confocal images do not allow a quantification of protein abundance, P_*METE*_ clearly drove much higher expression of the transgene than that of P_*CBA1*_, mirroring the RT‐qPCR experiments on the endogenous genes (Figure 1a). We decided therefore to focus on the characterisation of the former promoter in the rest of this study.

**Figure 2:**
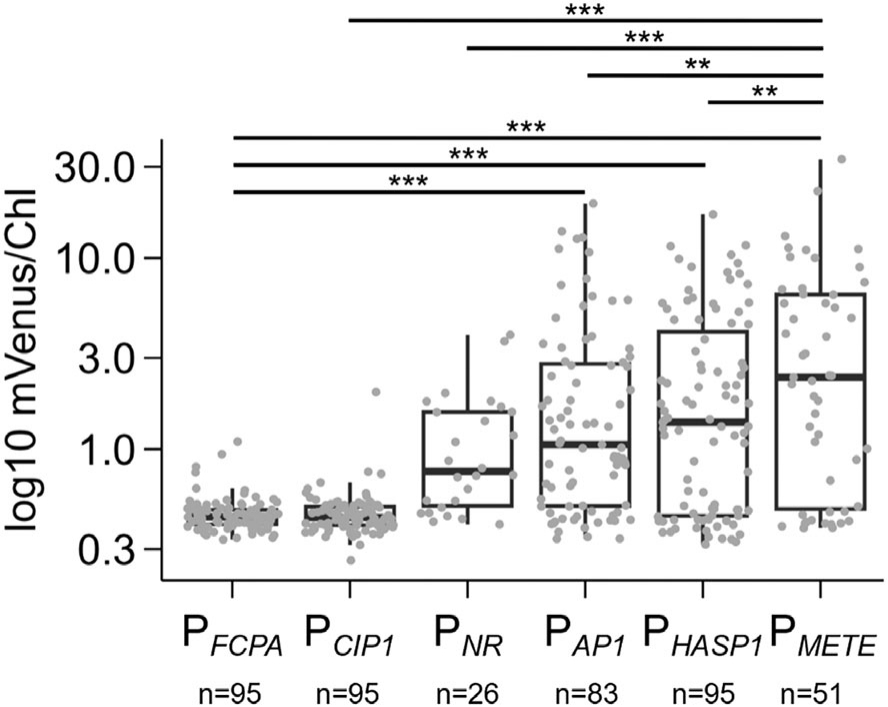
Comparison of different promoters driving mVenus expression in *P. tricornutum*. *P. tricornutum* was transformed with mVenus reporter constructs driven by different promoters. In addition to P_*METE*_ and P_*FCPA*_, these were the putative replication‐associated protein gene from the *Chaetoceros lorenzianus*‐infecting DNA virus (P_*CIP1*_), and the endogenous *P. tricornutum* promoters of nitrate reductase (P_*NR*_), alkaline phosphatase (P_*AP1*_), and highly abundant secreted protein 1 (P_*HASP1*_) (see text for details). Up to 95 independent transformants (*n*) of each were grown in 96‐well plates in standard f/2 media (i.e. 36 mm phosphate, no B_12_). Chlorophyll (Chl) and mVenus fluorescence was recorded on day 4. Each dot represents the mVenus signal normalised to chlorophyll fluorescence of an independent transformant. Statistical comparisons were made using a one‐way ANOVA and Tukey's multiple comparisons post‐hoc test. Significant differences are indicated above boxplots: ***P ≤ 0.001, **P ≤ 0.01.

First, we wanted to compare the expression of transgenes driven by P_*METE*_ with that by other characterised promoters. As well as P_*FCPA*_, we chose those from the endogenous genes *NR* (nitrate reductase; Poulsen and Kröger, 2005), *AP1* (alkaline phosphatase; Lin et al., 2017), and *HASP1* (highly abundant secreted protein 1; Erdene‐Ochir et al., 2019), as well as the *CIP1* promoter of the putative replication‐associated protein gene from the *Chaetoceros lorenzianus*‐infecting DNA virus (Kadono et al., 2015). As before, the promoter/5′UTR sequences (detailed in Table S3) were cloned in front of the *mVenus* reporter gene and transformed into *P. tricornutum* by electroporation. To allow a fair comparison of the relative strengths of the different promoters, 26–95 individual transformants were selected for each promoter construct, providing a large population from which to assess the promoter activity. Transformants were cultured in f/2 + zeocin in 96‐well plates for 7 days, then subcultured into fresh f/2 + zeocin and mVenus and chlorophyll fluorescence recorded on day 4. The mVenus values above background were normalized to chlorophyll to account for cell density variation across the plate (Figure 2). The lowest median values were recorded for lines transformed with the P_*FCPA*_ and P_*ClP1*_ constructs. No statistically significant difference was observed (Tukey's multiple comparisons post‐hoc test) between these sets of transformants, nor with those for P_*NR*_, although the mean was 2–3 fold higher and there was a wider range of values (note the log scale). The average mVenus/chlorophyll signal for lines transformed with either the P_*AP1*_ or P_*HASP1*_ constructs was 5–6 times higher compared with the P_*FCPA*_ lines and those for P_*METE*_ lines were approximately 10‐fold greater, all significantly different (*P* ≤ 0.001) from P_*FCPA*_. P_*METE*_ lines showed on average 1.8, 2, and 3.4‐fold higher mVenus/chlorophyll signal compared with P_*HASP1*_, P_*AP1*_, and P_*NR*_, respectively (*P* ≤ 0.01), hence being the strongest promoter in this experimental set‐up. For all four, there were several outliers with much higher expression, but the highest was still for a P_*METE*_ line, which had a signal 10 times that of P_*METE*_ mean and 70‐fold above that for P_*FCPA*_.

### Characterisation of transgene expression regulated by the *METE* promoter

**Figure 3:**
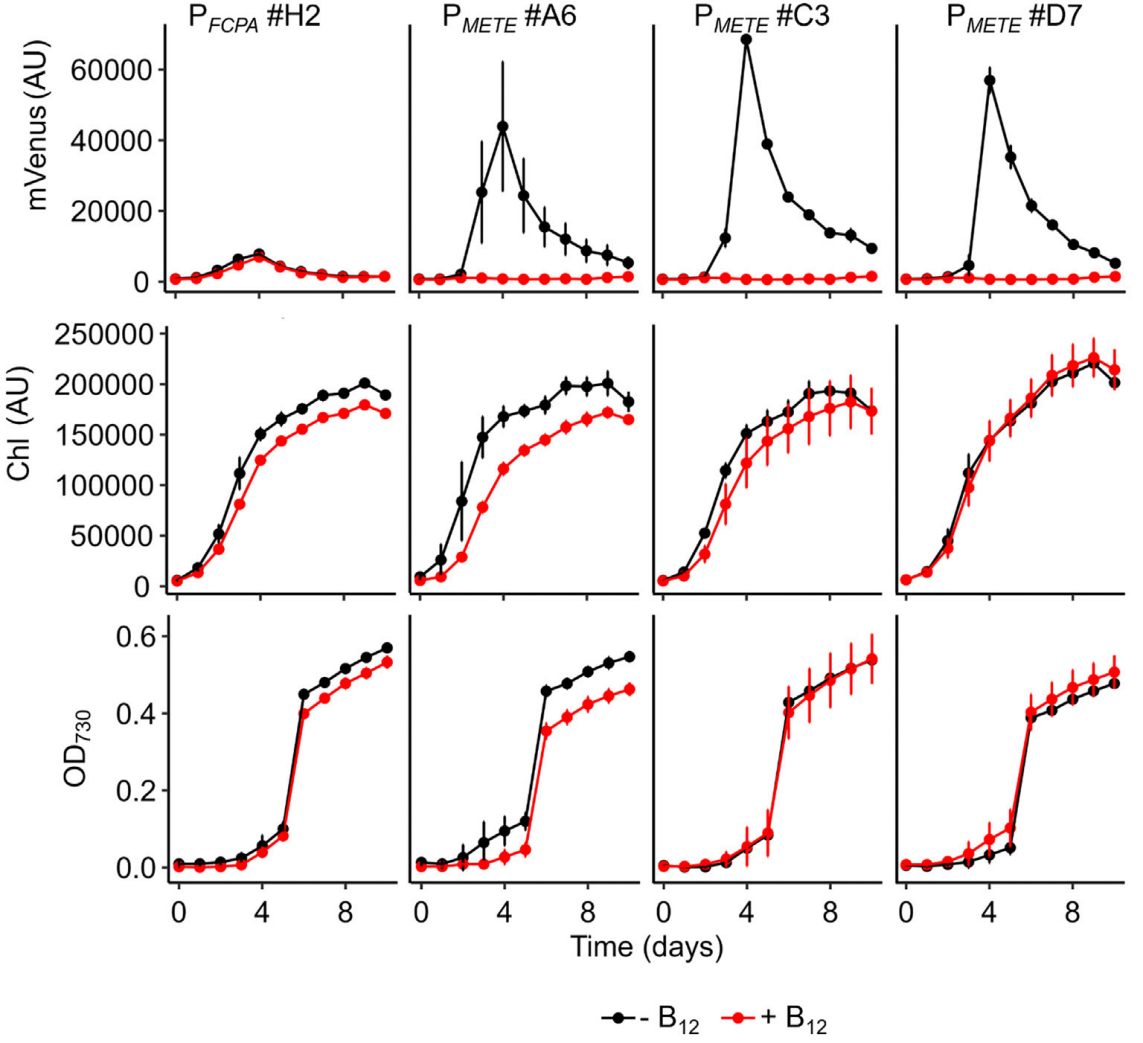
Time course of mVenus expression in reporter lines. Three representative transformants of P_*METE*_ (#A6, #C3, #D7) and one P_*FCPA*_ line (#H2) were grown in 96‐well plates in f/2 media in the absence of B_12_ (black symbols) or 1 μg L^−1^ B_12_ (red symbols). mVenus fluorescence, Chl fluorescence, and OD_730_ were recorded over time. Error bars represent the standard deviation of three biological replicates. AU, arbitrary units.

Three independent P_*METE*_ reporter lines exhibiting a high mVenus signal over several subcultures (subcultured every 7 days for 3 weeks) (#A6, #C3, #D7) were further investigated. First, we tested the time course of mVenus fluorescence over the culture growth, using one P_*FCPA*_ line for comparison (Figure 3). We also tested the effect of the addition of 1 μg L^−1^ B_12_. In the absence of B_12_, the P_*METE*_ lines exhibited an increase in mVenus fluorescence from day 0 until day 5, when fluorescence peaked and then gradually decreased for the remainder of the time course. A similar expression profile was observed for the P_*FCPA*_ line, although the signal was approximately 7–10 times less (*P* ≤ 0.001, Day 4). By regular subculturing at 3‐day intervals in fresh media so that the cells did not enter the stationary phase, the reporter gene activity could be maintained (Figure S3). The decrease observed could therefore mean either that the promoters are more strongly expressed when cells are in the log phase compared with the stationary phase (after day 6), or that there is quenching of the mVenus signal at higher cell densities.

**Figure 4:**
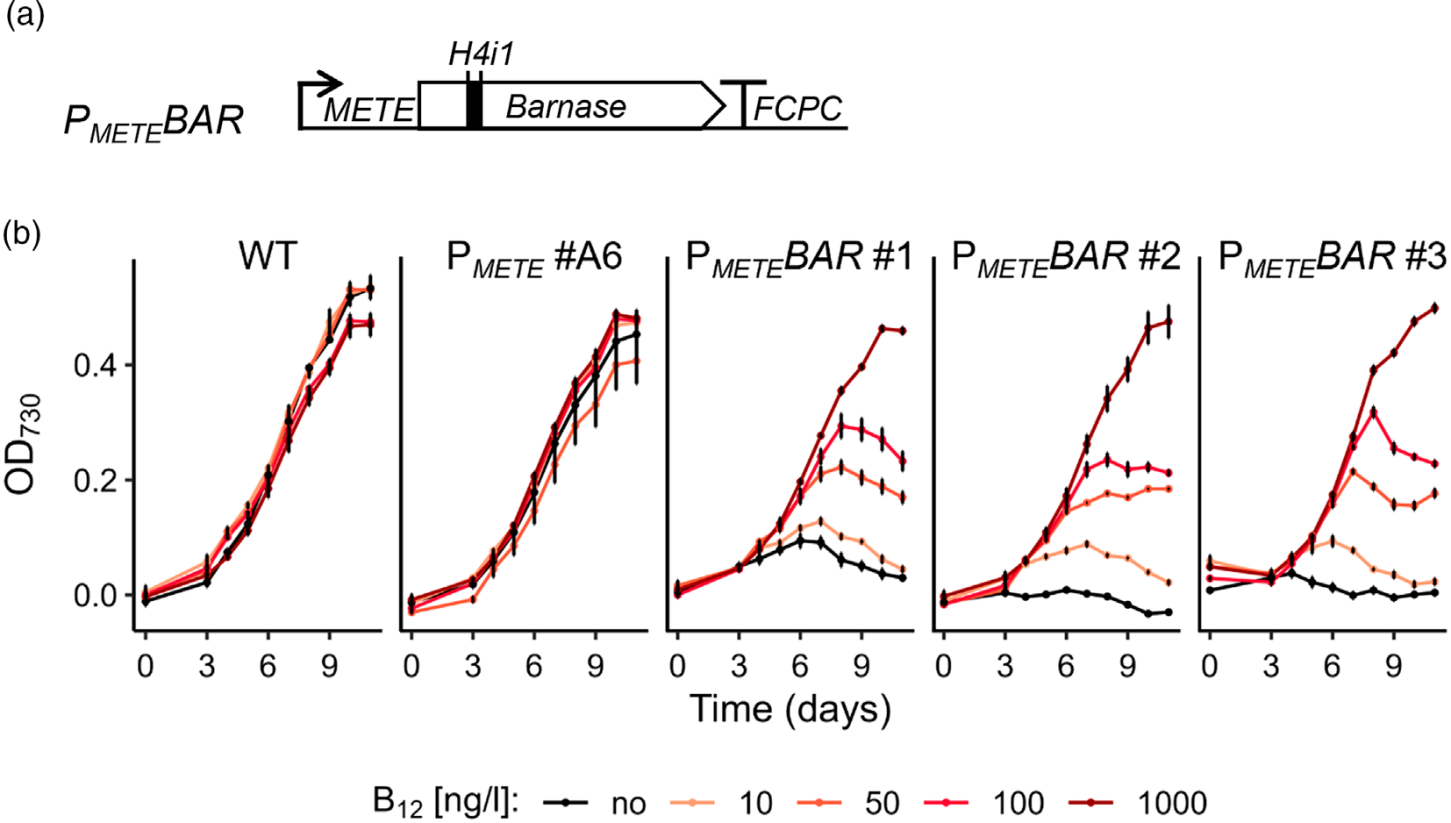
The *METE* promoter shows tight transgene regulation. (a) A schematic of the construct coding for the barnase enzyme under the control of P_*METE*_. The barnase coding sequence (542 bp) included an *H4* intron (84 bp) to prevent toxicity during cloning in *E. coli*. (b) Growth curves of *P. tricornutum* wild‐type (WT), a P_*METE*_ mVenus reporter line (#A6) and three independent transformants of P_*METE*_Bar (#1, #2, #3). Lines were cultured in f/2 without B_12_ (black) or at varying concentrations of B_12_ (orange to red), and OD_730_ was measured as a proxy for growth. Error bars represent the standard deviation of three biological replicates.

In lines grown with B_12_, no mVenus signal was recorded in P_*METE*_ lines, but the mVenus signal of the P_*FCPA*_ line was unaffected. Although in this experiment there was a slight reduction in chlorophyll fluorescence and OD_730_ for lines P_*FCPA*_ #H2 and P_*METE*_ #A6 when grown with B_12_, this was not always observed, particularly at lower concentrations of the vitamin (see for example Figure 4b). This is in contrast to the P_*AP1*_ promoter, where under the permissive conditions (no phosphate in the medium; Lin et al., 2017) a approximately 50% reduction in chlorophyll is seen in all lines and in some cases a considerable growth penalty (Figure S4). These data demonstrate that P_*METE*_ is a strong promoter that is responsive to the addition of a small molecule to the culture and under permissive conditions has no deleterious impact on the cells.

Reversibility of gene repression by B_12_ was investigated in an independent experiment where cells were grown in excess B_12_ (1 μg L^−1^) and then subcultured into fresh media with different concentrations of the vitamin. Monitoring mVenus expression over time resulted in some expression after just 18 h for cultures in no B_12_, followed by a rapid rise, and indeed expression returned to baseline in all cultures by 72 h (Figure S5). As seen previously, the signal declined steadily in stationary phase under all conditions, demonstrating that the promoter behaviour had been restored. The exception was the culture grown with 250 ng L^−1^ B_12_, which showed no fluorescence throughout the time course, reflecting the sensitivity of the promoter.

The possibility remains that some basal level of expression is present that is below the limits of detection of mVenus fluorescence. Accordingly, we used a lethality‐assay to investigate the extent to which the *METE* promoter is repressed by B_12_ by attempting to express the gene for the ribonuclease, barnase, which is lethal when expressed independently of its associated inhibitor Barstar (Hartley, 1988; Ramos et al., 2005). The barnase coding sequence from *Bacillus velezensis* was cloned downstream of P_*METE*_ (Figure 4a). Intron 1 from histone 4 (H4i1, Phatr3_J26896) was inserted into the coding sequence to prevent toxicity of the ribonuclease in *E. coli* and thus facilitate its cloning. As before, the *ble* gene was used to confer resistance to zeocin as the selectable marker. *P. tricornutum* cells were transformed using electroporation and plated on f/2 media with zeocin and 10 μg L^−1^ B_12_ to maximise repression, and up to 10 transformants were obtained. No transformants were obtained when B_12_ was omitted from the selection plates. In addition, no transformants were identified when *P. tricornutum* was transformed with cassettes utilising the *FCPA* promoter to drive the expression of the barnase enzyme, as would be expected since the protein would be produced constitutively and thus be lethal in transformants. Three independent P_*METE*_Bar transformants were picked and inoculated, along with controls of wild‐type and P_*METE*_ #A6, in 96‐well plates containing f/2 media supplemented with varying concentrations of B_12_. We recorded growth data (OD_730_), using culture viability as a proxy for the expression of barnase (Figure 4b). In the presence of 1 μg L^−1^ B_12_ (dark red lines), all transformants exhibited similar growth patterns, reaching stationary growth after approximately 10 days, indicative of a healthy culture. The wild‐type and P_*METE*_ reporter lines grew similarly under all tested B_12_ concentrations. In contrast, the three independent P_*METE*_Bar lines exhibited a decrease in growth, inversely proportional to the concentration of the vitamin present, indicating that the barnase enzyme was correctly translated and active within the cells. Our results thus demonstrate that the *METE* promoter has a tight ‘off’ characteristic in the presence of B_12_ above a threshold of between 100 and 1000 ng L^−1^ B_12_.

### Effect of the *METE* terminator and promoter truncations on transgene expression driven by P_*METE*_

**Figure 5:**
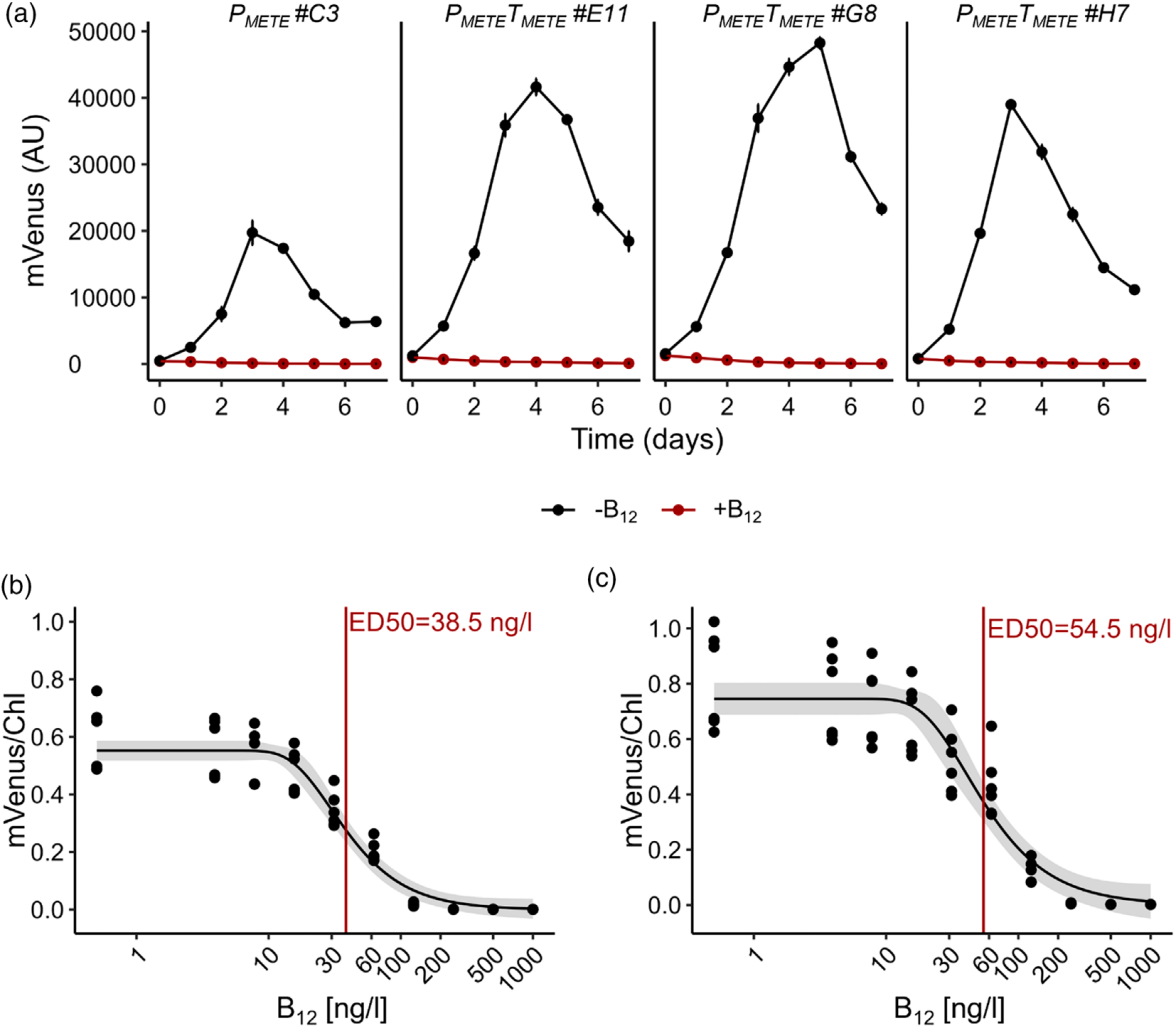
The *METE* terminator influences transgene expression levels but not B_12_‐dependent tuneability. (a) Three representative transgenic lines (#E11, #G8, #H7) that had been transformed with a construct that included the *METE* terminator (P_*METE*_T_*METE*_) were grown over time as before, and mVenus fluorescence was monitored. As a reference, the P_*METE*_ line #C3 harbouring the *FCPC* terminator (see Figure 3) was analysed simultaneously. Error bars represent the standard deviation of three biological replicates. (b) B_12_‐dependent dose–response in *P. tricornutum* lines expressing mVenus under control of the *METE* promoter and *FCPC* terminator. Normalised fluorescence (mVenus/Chl) at day 4 is plotted against various B_12_ concentrations (no B_12_, 4–1000 ng L^−1^). (c) As for (b) but with P_*METE*_ T_*METE*_ lines. The drc package with Weibull parameters was used to quantify the effective dose (ED, Ritz et al., 2015). Vertical line marks the ED50, which is responsible for repressing 50% of the mVenus reporter for (b) of 38.5 ng L^−1^ B_12_ in P_*METE*_ lines and (c) of 54.5 ng L^−1^ B_12_ in P_*METE*_T_*METE*_ lines.

From work in *C. reinhardtii* and other algae, it is known that transgene expression is not only influenced by its specific promoter and 5′UTR, but also by the corresponding 3′UTR and terminator (Geisler et al., 2021; Kumar et al., 2013). We therefore tested a construct using the 400 bp long *METE* 3′UTR and terminator (hereafter T_*METE*_, Figure S1) instead of the T_*FCPC*_ in a mVenus expression cassette driven by P_*METE*_. Obtained transformants were tested for mVenus expression, and three independent P_*METE*_T_*METE*_ lines were further analysed in parallel to the previously tested P_*METE*_ #C3 (with T_*FCPC*_, Figure 3). Lines transformed with the P_*METE*_T_*METE*_ construct (#E11, #G8, #H7) showed approximately a 2.0 to −2.5‐fold higher overall mVenus fluorescence in the absence of B_12_ compared with the P_*METE*_ #C3 (Figure 5a). Adding B_12_ (1 μg L^−1^) resulted in repression of the mVenus signal in all lines. This shows that the inclusion of the native *METE* terminator instead of the *FCPC* terminator improves protein expression levels in reporter constructs driven by the *METE* promoter. This improvement was confirmed in an RT‐qPCR experiment measuring steady‐state levels of mVenus transcript (Figure S6).

To quantify the dynamic range of the B_12_‐dependent response in more detail, an independent experiment was performed growing lines in f/2 media containing either no B_12_ or a range of different B_12_ concentrations (4–1000 ng L^−1^ B_12_) and recording mVenus and chlorophyll fluorescence (Figure 5b,c). Plotting the normalised mVenus fluorescence signal against an increasing B_12_ concentration allowed calculation of the effective dose (ED50) required to repress 50% of the transgene (Ritz et al., 2015). ED50 values on day 4 were similar, at 38.5 and 54.5 ng L^−1^ for P_*METE*_ and P_*METE*_T_*METE*_ respectively, and complete repression of the mVenus signal was seen at 250 ng L^−1^ for both constructs. This indicates that the terminator has no effect on the B_12_‐responsiveness.

**Figure 6:**
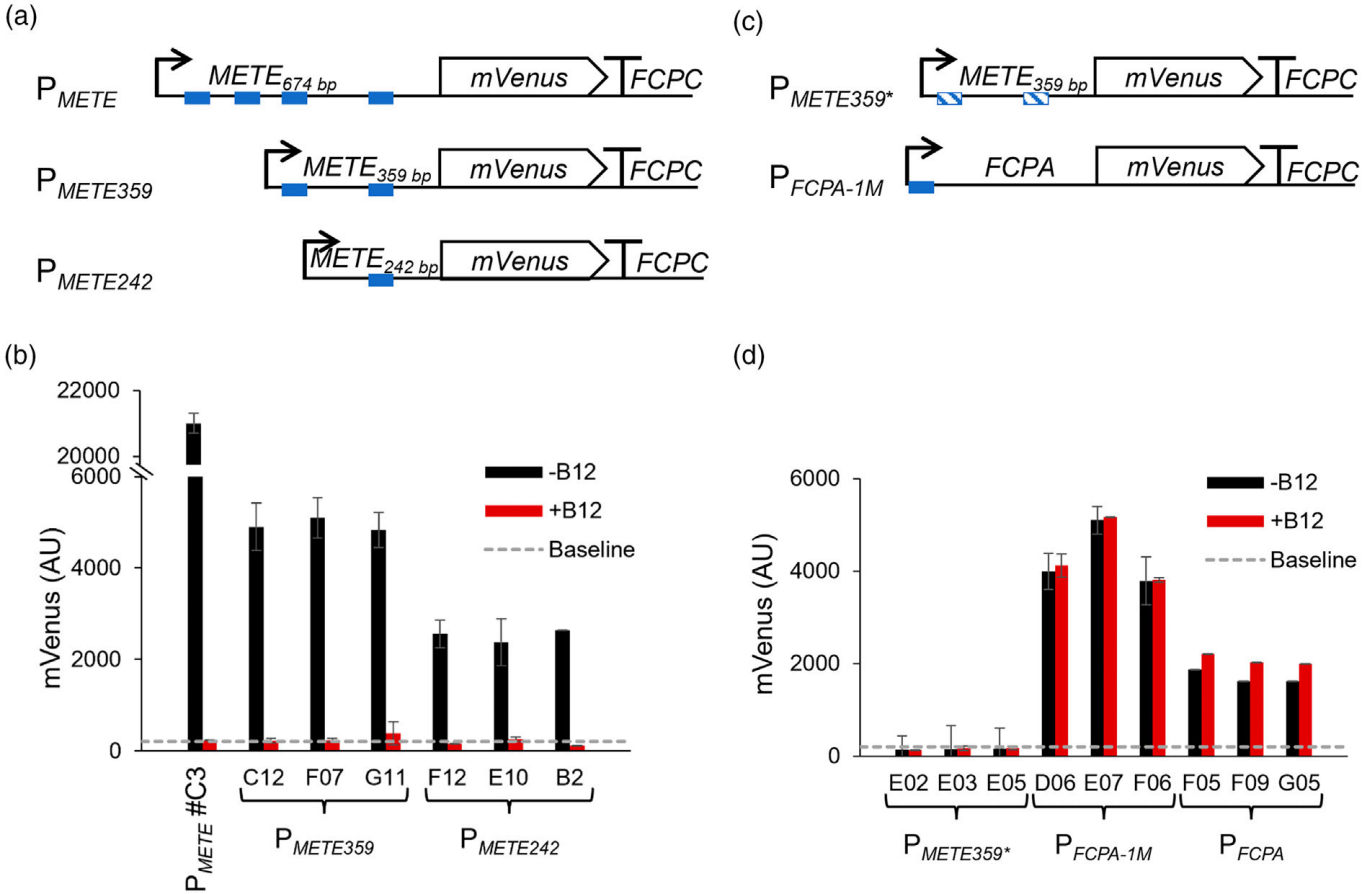
*METE* promoter and 5′UTR truncations influence transgene expression levels but not B_12_‐dependent regulation. (a) Schematics of *METE* promoter truncation constructs. The length of the *METE* promoter/5′UTR used in the constructs is indicated in base pairs (bp) from the start codon. The location of an identified 14 nt motif sequence (5′‐GAAKYACGTKCWKC, Figure S6) is indicated by blue squares. All constructs were assembled with an mVenus coding sequence and *FCPC* terminator. (b) Three representative transgenic lines transformed with P_*METE359*_ or P_*METE242*_ were grown in f/2 without B_12_ (black) or with 1 μg L^−1^ B_12_ (red). The mVenus fluorescence signal was recorded on day 4. As a reference, the P_*METE*_ line #C3 (see Figure 3) was analysed in the same experiment. Error bars represent the standard deviation of biological replicates (*n* = 3). The background fluorescence of untransformed cells is shown as a dashed grey line. (c) Schematic of construct P_METE359*_ encoding a truncated P_*METE*_ version that is similar to P_*METE359*_, but the two putative 14 nt motifs have been scrambled (dashed boxes). In plasmid P_*FCPA‐1M*_, one copy of the identified motif was cloned upstream of the *FCPA* promoter. (d) Three representative transgenic lines transformed with P_METE359*_ or P_*FCPA‐1M*_ alongside *P*_*FCPA*_ as control were analysed as in (b).

We speculated that the B_12_‐dependent regulation would be based on the presence of cis‐acting regulatory element(s) in the *METE* promoter. We therefore generated promoter truncations to test this. Constructs of 359 bp (1/2 of the full length) and 242 bp (1/3 of the full length) from P_*METE*_ were generated (Figure 6a) and used to transform *P. tricornutum* as before. Several transformants of P_*METE359*_ and P_*METE259*_ were screened and the three highest mVenus‐expressing lines were analysed further. These drove approximately 25% and approximately 10% of the original expression levels observed in P_*METE674*_ line #C3 respectively (Figure 6b). However, transgene expression was still completely repressed by 1 μg L^−1^ B_12_ in both truncated promoter versions. This suggests that the genetic element responsible for B_12_‐dependent regulation of P_*METE*_ is within the 242 bp of the ATG start codon, and that possibly the region upstream of 242 bp contains enhancer elements for expression.

We therefore further investigated the presence of conserved motifs in B_12_‐regulated promoters using the MEME motif discovery software (Bailey et al., 2009). We used as training input the promoter region of another B_12_‐regulated gene, *THIC* (Llavero‐Pasquina et al., 2022), encoding an enzyme of thiamine biosynthesis, 4‐amino‐5‐hydroxymethyl‐2‐methylpyrimidine pyrophosphate (HMP‐PP) synthase. Using 500 bp upstream from the start codon of *THIC* genes from six diatom species, *P. tricornutum, Fragilariopsis cylindrus, Pseudonitzschia multistrata, Thalassiosira pseudonana, T. oceanica* and *Cyclotella cryptica* (Table S4, Figure S7a,b), the algorithm identified a 14 nt motif, 5′‐GAAKYACGTKCWKC‐3′ (where K stands for G or T, Y for C or T, and W for A or T following the standard IUPAC nomenclature) with an e‐value of 670. The 5′ and 3′ flanking regions showed no identity. To validate whether the motif could be associated with B_12_ regulation more broadly, we subsequently searched for the motif in six other genes differentially regulated by B_12_ in diatoms that were not used in the training set for the MEME algorithm, including the three studied here from *P. tricornutum*, *METE*, *CBA1* and *SHMT1*. Five out of the six promoters tested contained the motif (Figure S7a,b). In contrast, the motif was not observed in eight other *P. tricornutum* genes known to be unaffected by B_12_ (gene function and ID Table S4). Subsequently, we combined all motif hits and generated a consensus motif of 5′‐GAAGCACGTGCTTC‐3′ (WebLogo3, Figure S7c).

Most of the regulated genes had one copy of the motif, whereas P_*METE674*_ had four, the truncated promoter P_*METE359*_ contained two motifs, and *P*_*METE259*_ had just one motif (shown as blue boxes in Figure 6a). To test whether the identified motifs were necessary for B_12_‐dependent regulation and/or gene expression, a version of the truncated P_*METE359*_ sequence was generated where both 14 nt motifs were randomly scrambled (P_METE359*_, Figure 6c). After transformation of these constructs into *P. tricornutum*, three positive colonies for each construct were grown in f/2 media with no or 1 μg L^−1^ B_12_, and mVenus fluorescence was measured on day 4 as before. Lines transformed with P_METE359*_ containing the scrambled 14 nt motifs exhibited no detectable mVenus fluorescence under either condition (Figure 6d). This observation would suggest that the motifs are essential for gene expression, but it was not possible to determine if it influenced B_12_‐dependent regulation. Conversely, a reciprocal construct, where the motif was added to the 5′ end of the constitutive 442 bp *FCPA* promoter to make P_*FCPA‐1M*_, resulted in transformants that all expressed mVenus but showed no B_12_‐dependent changes in expression levels. Moreover, the addition of one copy of the 14 nt motif to the *FCPA* promoter (P_FCPA‐1M_ lines) resulted in a approximately 2.3 times increase of mVenus signal compared with the lines only carrying the *FCPA* promoter (*P* < 0.01), suggesting that the 14 nt motif works as an enhancer of transgene expression, but alone it is not sufficient to mediate B_12_‐regulation.

### B_12_ ‐dependent production of a plant diterpenoid in *P. tricornutum*

**Figure 7:**
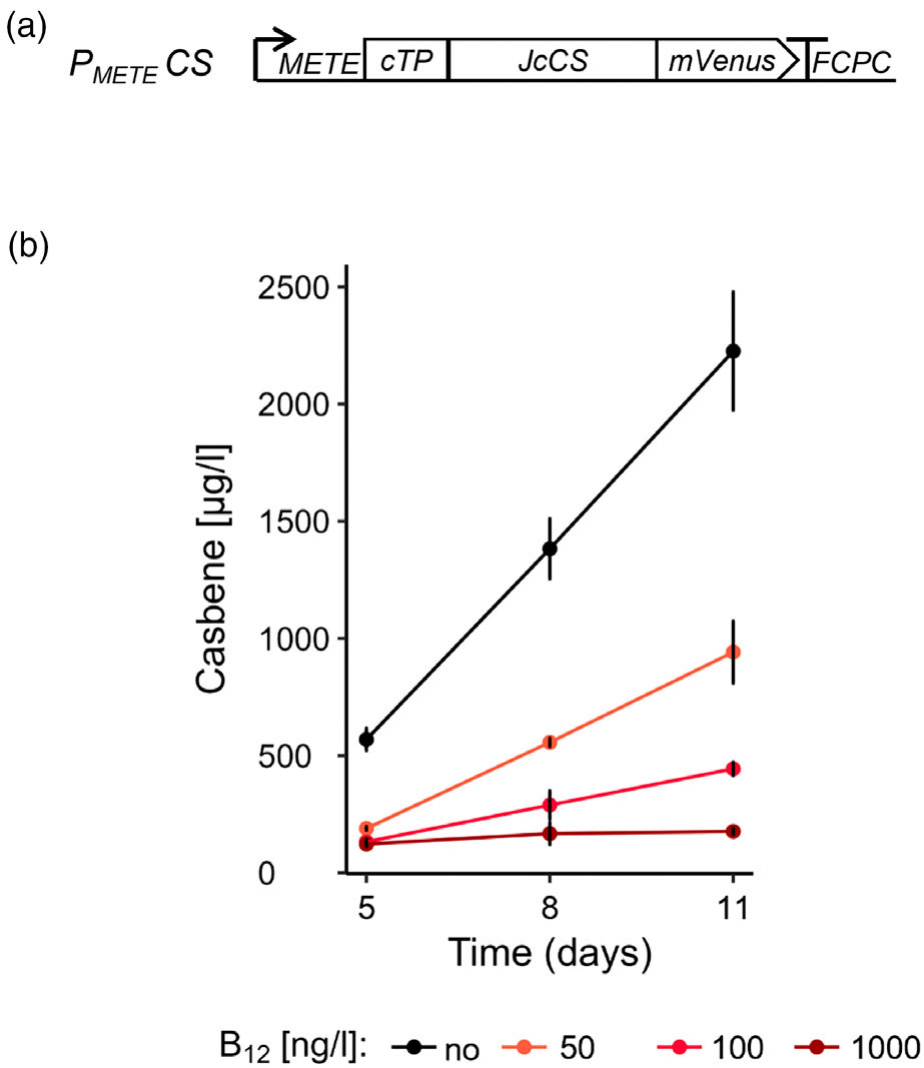
Casbene production driven by *METE* promoter. (a) Schematic of the P_*METE*_ *CS* expression cassette showing the full‐length version of the *METE* promoter driving the expression of casbene synthase from *Jatropha curcas* (JcCS) with a chloroplast target peptide (cTP) and mVenus C‐terminal tag. (b) The P_*METE*_ *CS* line #F05 was grown in 20 ml cultures in f/2 without B_12_ (black) or with various B_12_ concentrations (orange to red) and a dodecane overlay was added 2 days post‐inoculation. The dodecane overlay was analysed on days 5, 8, and 11 post‐inoculation by GC–MS to monitor casbene production. Error bars represent the standard deviation of three biological replicates.

*P. tricornutum* has been used as a production chassis for a range of high‐value compounds, including geraniol, a monoterpene (C10 branched hydrocarbon) (Fabris et al., 2020; George et al., 2020) and the C30 triterpene lupeol (D'Adamo et al., 2019). To investigate the effectiveness of the *METE* promoter in metabolic pathway engineering, we deployed it to regulate the heterologous production of the diterpene, casbene (C20). Casbene is produced from the precursor geranylgeranyl pyrophosphate (GGPP) by the bi‐functional enzyme casbene synthase (CS). It can be further modified to yield medically relevant diterpenoids such as jolkinol‐C (Vasas and Hohmann, 2014). Casbene has been produced in the green alga *C. reinhardtii* by overexpressing CS from *Ricinus communis* (Lauersen et al., 2018) or *Jatropha curcas* (Mehrshahi et al., 2020). We followed a similar strategy and assembled a construct containing the *J. curcas CS* coding sequence (*JcCS*) under the regulation of P_*METE*_ (Figure 7a). GGPP is the product of the methyl‐D‐erythritol phosphate (MEP) pathway in the chloroplast. To ensure correct localisation, the chloroplast targeting peptide from 1‐deoxy‐d‐xylulose 5‐phosphate reductoisomerase (DXR, *Phatr3_J9258*) was included upstream of the CS coding sequence (Table S3). A C‐terminal mVenus tag was also included to assist in the identification of high‐expression lines.

The P_*METE*_ *JcCS* construct was introduced into *P. tricornutum* cells via biolistic transformation, and several zeocin‐resistant transformants were picked and grown in a 96‐well plate in 200 μL f/2 media. Four days after subculturing, transformants were screened for mVenus fluorescence. Biolistic transformation can result in random fragmentation of plasmid DNA (Hopes et al., 2016) and wider genome rearrangements. In the context of a biotechnology application, we were screening for the highest mVenus expressing lines, as these should also be the highest casbene expressing lines. Six independent lines showing high mVenus fluorescent signals were chosen and grown as 2 ml cultures in a 24‐well plate. At day 2, 200 μL dodecane (10% v/v) was added as an overlay to each culture to sequester casbene being produced by the cells (Lauersen et al., 2018). After 5 days (i.e., 7 days post‐inoculation), the casbene content of the dodecane overlay was quantified using GC–MS (Mehrshahi et al., 2020). All six P_*METE*_ *JcCS* lines accumulated between 500 and 1000 μg casbene L^−1^ culture (Figure S8).

The P_*METE*_CS line #F05 was selected for further analysis and used to inoculate 20 ml cultures in a range of B_12_ concentrations. A 10% (v/v) dodecane overlay was added to each culture on day 2 and samples of dodecane were taken every 3 days thereafter (5, 8 & 11 days post‐inoculation) for GC–MS analysis (Figure 7b). In cultures that contained no B_12_, casbene levels rose from 500 μg L^−1^ on day 5 to almost 2500 μg L^−1^ casbene on day 11. In cultures with 50 and 100 ng L^−1^ B_12_, casbene was still detected and increased over the time course, albeit at reduced levels (up to ~940 and ~440 μg L^−1^ casbene at day 11, respectively). This is consistent with data we obtained for lines expressing mVenus under the control of the *METE* promoter (Figure 5c) where 50–100 ng L^−1^ B_12_ partially repressed transgene expression. Casbene was detected (~150 μg L^−1^) in cultures grown in media containing 1 μg L^−1^ B_12_, but did not increase over time. In this case, it is likely that the cells used for inoculation, which were grown without B_12_ and so would have been actively transcribing the *JcCS* gene at the point of inoculation, retained some translated CS protein, whose activity was present during the remainder of the experiment.

In diatoms and other photosynthetic organisms, GGPP is the precursor for the photosynthetic pigments, chlorophyll and carotenoids. Overexpressing CS in the chloroplast of *P. tricornutum* might therefore influence the available pool of GGPP, so we investigated whether the presence of a functional CS in the chloroplast would draw precursors from the biosynthesis of pigments. We analysed carotenoids and chlorophylls (*a* and *c*) in the representative P_*METE*_ *JcCS* #F05 line at the end of the dodecane overlay experiment. In media containing no B_12_, cells had lower levels of carotenoids than those grown with the vitamin (Figure S9a), although this was not statistically significant. No difference in chlorophyll levels was observed between the different conditions (Figure S9b), even though cultures containing B_12_ did not produce casbene (Figure 7b). As a result, we conclude that the GGPP pool was not depleted in the cells where *JcCS* is expressed. This experiment highlights that the use of a strong tuneable promoter can help understand and engineer metabolic pathways in *P. tricornutum*.

## DISCUSSION

In this study, we have demonstrated that the promoter from the *P. tricornutum METE* gene is a valuable tool for genetic manipulation of this versatile biotechnological chassis. Comparing the strength of promoters across studies can be problematic because different growth conditions can influence transgene expression levels, so to obtain a quantitative readout, we measured reporter gene expression in transformants using identical constructs, grown in the same media and at the same time (day 4 after inoculation). Several independent transformants (*n* ≥ 50 in Figure 2) were analysed to circumvent potential bias of positional transgene integration (George et al., 2020; Peach and Velten, 1991). The results showed that P_*METE*_ is one of the strongest promoters that have been reported for this microalga under the conditions assessed in this study. Several recent studies have found that the promoter of gene *Phatr3_J49202* can drive strong gene expression (Fabris et al., 2020; Garza et al., 2023). *Phatr3_J49202*, which encodes a protein of unknown function, is consistently observed to be one of the most highly expressed genes in transcriptomic data sets from *P. tricornutum*. Like P_METE_, strains transformed with protein‐mVenus fusion proteins under the control of P_*Phatr3_J49202*_ actively express the protein during most of the cultivation with a peak expression during the exponential phase (Fabris et al., 2020). P_*METE*_ has the added advantage of being tightly regulated by nanomolar levels of a benign compound, vitamin B_12_ (cobalamin), in a reversible and tunable fashion.

Maximal expression with P_*METE*_ was observed during the log phase, with a decline as the culture entered the stationary phase (Figure 3). This was also seen with P_*FCPA*_ and P_*AP1*_ (Figure S4) and most other characterised *P. tricornutum* promoters. The exception was the *HASP1* promoter, which has been demonstrated to exhibit strong activity during all growth phases, especially during the stationary phase (Erdene‐Ochir et al., 2019). We found that culturing *P. tricornutum* lines in a semi‐continuous manner, so that the cells were artificially maintained in an exponential growth phase, allowed for high transgene expression throughout cultivation (Figure S3).

Transgene expression levels can also be influenced by genetic elements other than the promoter, including introns (Baier et al., 2018; Eichler‐Stahlberg et al., 2009), the 5′UTR (Nguyen et al., 2009), and the 3′UTR/terminators (e.g. Geisler et al., 2021; Kumar et al., 2013). In our study, we initially tested all promoters with the *FCPC* terminator. We then combined *P*_*METE*_ with its cognate terminator and found that the mVenus fluorescence was increased by a factor of two (Figure 5). Moreover, RT‐qPCR analysis showed higher steady‐state transcript levels of the reporter gene than the endogenous *METE* gene in the same line (Figure S6), whether or not the terminator was included, suggesting that removal from the endogenous genomic context removed one or more repressive elements. Nevertheless, B_12_‐dependent regulation of the reporter gene was not influenced and remained tuneable with ED50 values of 40–50 ng L^−1^ (Figure 5b,c), the same order of magnitude as seen for the *METE* gene itself (~100 ng L^−1^; Helliwell et al., 2014).

As well as high levels of activity, P_*METE*_ is more versatile than other tuneable endogenous promoters, such as P_*AP1*_ or P_*AMT1*_, since it has no deleterious effects under the permissive condition and few other genes are influenced by the presence or absence of the ligand (on average 6%, Bertrand et al., 2012) (Figure 3 and Figure S3). Moreover, extremely low concentrations of vitamin B_12_ (250 ng L^−1^, equivalent to ~200 pM) are sufficient to suppress transgene expression by >90% (Figure 5). This would offer cost advantages in large‐scale cultivation. Similar efficacy has been observed in *P. tricornutum* using synthetic rather than native expression systems, DIG/pUAS and XVE/OlexA (Kassaw et al., 2022). Linear induction of the mVenus reporter was observed as levels of digoxin were raised to 100 μM for DIG/pUAS and up to 1 μM β‐oestradiol for XVE/OlexA, although higher levels of β‐oestradiol caused a longer lag phase in cultures. The dynamic range for P_*METE*_ was >100 fold (Figure 3), comparable to that of approximately 90 and 180‐fold for DIG/pUAS and XVE/OlexA, respectively.

From a biotechnology standpoint, keeping tuneable promoters in the ‘off’ state can minimise the interference of gene products or their metabolites with normal cellular processes, thereby maximising biomass generation prior to induction. The *METE* promoter provides extremely tight regulation of transgene expression, demonstrated by no detectable mVenus fluorescence above 250 ng L^−1^ B_12_, but even more so by the use of the barnase system (Figure 4), where even very low levels of barnase expression are highly cytotoxic (Edelweiss et al., 2008) and have been used widely to test the extent of repression in other systems (e.g. Shankar et al., 2021). As well as for metabolic engineering, the *METE* promoter would be a powerful tool to study potential lethal phenotypes of native gene knockouts.

To understand the regulatory mechanism for and to optimise use of the B_12_‐repressible module in synthetic biology constructs, identifying the minimal DNA sequence to confer B_12_‐dependent expression is important. Yoshinaga et al. (2014) investigated the regulation of iron‐responsive *iron starvation induced protein1* (*Isi1*), *ferrichrome binding protein1* (*FBP1*), and *flavodoxin* (*Fld*) using promoter truncations of the three promoters to express a β‐glucuronidase reporter gene. They identified core regions responsible for the response to iron limitation and demonstrated that a 34 bp iron‐responsive element of the *FBP1* promoter cloned upstream of the constitutive cytomegalovirus promoter (P_*CMV*_) was able to confer an iron‐dependent response to this promoter (Yoshinaga et al., 2014). By comparing several promoters previously known to be downregulated by B_12_, we identified a common 14 nt motif, with four copies of the motif found in P_*METE*_ (Figure S7, Table S4). We found experimentally with promoter truncations that reduced copy number of the motif, or mutagenesis of the motif sequence, that it was necessary for gene expression but not for B_12_‐dependent regulation (Figure 6). When the motif was added to the low‐expressing *FCPA* promoter, it boosted expression, but no B_12_‐dependent regulation was observable. This enhancer function of the motif likely corresponds to a transcriptional activator, examples of which have been shown to be repressible by metabolites such as sulphur and glucose (Carlson, 1999; Maruyama‐Nakashita et al., 2006; Tao and Marzluf, 1998). The palindromic nature and the CACGTG (G‐Box) at the centre of the motif suggest it is bound by a protein containing a bHLH or a bZIP DNA‐binding domain (Ezer et al., 2017; Llavero‐Pasquina, 2020). Yeast 1 Hybrid (Y1H) or electrophoretic mobility shift assays (EMSA) experiments could be used to test whether candidate proteins containing bHLH or bZIP domains in *P. tricornutum* bind the motif.

We engineered *P. tricornutum* to produce the diterpenoid casbene by the introduction of a casbene synthase gene under the *METE* promoter, so that casbene production could be tuned by the addition of B_12_. In the absence of B_12_, a titre of approximately 2.1 mg casbene L^−1^ was achieved in a 20 ml culture after 11 days (~0.6 mg casbene L^−1^ after 5 days, ~1.4 mg casbene L^−1^ after 8 days, Figure 7). This is similar to those reported in the green alga *C. reinhardtii*: using different promoters to drive the expression of the *Ricinus communis* casbene synthase, the titre varied between ~1 (P_*βTUB2*_) and ~ 3.5 (P_*PSAD*_) mg L^−1^ (Einhaus et al., 2021). Mehrshahi et al. (2020) obtained 2 mg L^−1^ using P_*PSAD*_, but in addition regulated casbene levels by the use of a thiamine‐dependent riboswitch, which allowed casbene production only in the absence of thiamine. Other studies using *P. tricornutum* for terpenoids produced measurable amounts, but lower than reported here. For example, D'Adamo et al. (2019) were able to produce 0.1 mg L^−1^ lupeol over 2 days in *a P. tricornutum* strain expressing lupeol synthase under the control of the constitutive *FCPA* promoter, and expression of the monoterpene synthase, geraniol synthase under the control of the *Phatr3_J49202* promoter resulted in a titre of 0.309 mg L^−1^ geraniol after 7 days under batch conditions (Fabris et al., 2020); the *Phatr3_J49202* promoter had been identified by looking for genes that are highly expressed when *P. tricornutum* was grown in batch cultures. It should be noted that in the two latter examples, the terpenoids were extracted at an endpoint measurement, while in the study presented here, casbene was captured with a dodecane overlay, allowing a continuous accumulation of casbene.

Casbene is of potential commercial interest as an antifungal agent, but it is also the precursor to a range of bioactive jolkinols and ingenates (King et al., 2014; Vasas and Hohmann, 2014). To synthesise these compounds, it is likely necessary to achieve a high flux from the precursor GGPP to casbene (Forestier et al., 2021). This could be achieved by upregulation of the synthesis of the precursor GGPP, as has been shown for the production of the diterpenoid sclareol in *C. reinhardtii* (Einhaus et al., 2022). Together with optimization of growth conditions and media composition, manipulation of the endogenous metabolic pathways led to a substantial increase in sclareol up to 656 mg L^−1^, a similar order of magnitude to those achieved by engineering the pathway in yeast (750 mg L^−1^; Trikka et al., 2015) or *E. coli* (1.5 g L^−1^; Schalk et al., 2012). However, it was observed that high levels of diterpenoid production impacted the health of the *C. reinhardtii* culture (Einhaus et al., 2022). Under these circumstances, the tuneable nature of *P*_*METE*_ offers a simple solution, allowing repression of the branchpoint enzyme – the diterpenoid synthase in this case – until sufficient biomass has been produced. With an appropriate dosing regimen, levels of B_12_ can be controlled simply by dilution within the culture, allowing gradual induction of the pathway over a period of a few days.

Increasingly, the potential of engineering biology is being harnessed to generate novel products and processes of commercial value, using the versatility of microbial metabolism and their ability to be cultivated at scale under optimal conditions to maximize yield and reduce costs (Liu and Nielsen, 2019). A key issue is the requirement for carbon sources as feedstocks for the cultivation, which can impact both the cost of the process and the overall sustainability. The photosynthetic nature of microalgae such as *P. tricornutum* offers an alternative approach (Russo et al., 2023), with the potential to be more carbon neutral than using heterotrophic microorganisms such as bacteria or yeasts (Kazamia and Smith, 2014; Moses et al., 2017). To be able to compete with these conventional chassis, sophisticated strategies for genetic manipulation of algae are required. The P_*METE*_ will be an important contribution towards developing *P. tricornutum* as an industrial biotechnological platform.

## MATERIAL AND METHODS

### Algal strains, growth conditions and transformation

*P. tricornutum* strain CCAP 1055/1 was grown in f/2 medium at 18°C and 60–80 μmol photons m^−2^ sec^−1^ under a 16 h light/8 h dark cycle. If required, antibiotics and vitamin B_12_ (cyanocobalamin; Sigma‐Aldrich, Dorset, UK) were added to the medium at indicated concentrations.

For transformation, *P. tricornutum* cells were grown in f/2 minus silica medium to an early exponential growth phase (~2–4·10^6^ cells ml^−1^). For the majority of the experiments, cells were transformed with plasmids using a NEPA21 Type II electroporator (Nepa Gene) as previously described (Yu et al., 2021) (Figures 1, 2, 3, 4, 5, 6 and Figures S2– S6). Cell lines transformed with a construct encoding casbene synthase were generated by biolistic transformation (Figure 7). For transformation by bombardment, DNAdel™ 500 nm gold particles (Critter Technologies, San Diego, CA, USA) were coated in plasmid DNA at a concentration of 2.5 μg mg^−1^ gold particles according to the manufacturer's instructions and resuspended in 100% ethanol to a final concentration of 50 mg mL^−1^ DNA‐coated gold particles. Bombardment was carried out using a Biolistic® PDS‐1000/He (Bio‐Rad, Watford, UK) with a 1350 psi rupture disc (Bio‐Rad). A vacuum was applied to the chamber and particles were fired at a pressure of 1550 psi.

After electroporation or bombardment, cells were incubated for 24 h at 18°C under constant light before cells were transferred to 1.5% f/2‐agar selection plates containing 75 μg ml^−1^ zeocin (InvivoGen Europe, Toulouse, France) and incubated for 2–3 weeks. Zeocin‐resistant colonies were picked into 96‐well plates (Starlab, Milton Keynes, UK) containing 200 μL of f/2 media with 75 μg mL^−1^ zeocin and sub‐cultured every 7 days before further analysis.

### Analysis of gene expression by quantitative PCR

Wild‐type *P. tricornutum* was grown to early stationary phase in f/2 media. On day 7, the cultures were centrifuged, and the cell pellet was resuspended in 30 ml f/2 with no B_12_ or 1 μg L^−1^ B_12_. After 24 h, the cells were pelleted and snap frozen in liquid nitrogen. RNA was extracted using Qiagen RNeasy^®^ Plant Mini Kit (Hilden, Germany). The cell pellet (~30 g fresh weight/column) was initially resuspended in the lysis buffer plus 300 μL zirconia beads (Sigma‐Aldrich) and vortexed for 15 min at 4°C. After centrifugation, the supernatant was processed according to the manual, except that the column was washed four times instead of two times before eluting the RNA. DNA was removed with one unit (U) Turbo DNA‐free™ kit (Thermo Fischer Scientific, Paisley, UK) for 30 min. cDNA synthesis was carried out using random hexamers as primers and following the manufacturing instructions provided in the SuperScript^®^III First‐Strand synthesis system for RT‐PCR Kit (Invitrogen, Paisley, UK). The SYBR^®^ Green JumpStart™ Taq ReadyMix™ (Sigma‐Aldrich) was used for quantitative PCR with CFX384 Touch Real‐Time PCR Detection System (Bio‐Rad), with 40 cycles of 20 sec at 94°C, 20 sec at 55°C, 30 sec at 72°C. Transcript levels were normalised to housekeeping gene *ubiquitin conjugating enzyme* (*UBC*; *Phatr3_J28433*) or *histone H4* (*H4*, *Phatr3_J26896*), which had the most accurate efficiency with relative expression calculated according to the ΔΔCT comparative quantification method (Schmittgen and Livak, 2008). Primer efficiencies were validated to be able to compare qPCR values between different primer pairs (Pfaffl, 2001) and Table S2 provides details of primers.

### Plasmid design and assembly

Standard molecular biology methods for PCR and plasmid purification were carried out using reagents from New England Biolabs (NEB, Hitchin, UK) or Thermo Fisher Scientific. 5‐alpha competent *E. coli* cells (NEB) were used for cloning purposes. Constructs were made using parts encoding promoters, 5′UTRs, and terminators from *P. tricornutum* genomic DNA, cDNA, and in‐house plasmid templates for reporter genes and selectable markers generated by the Smith laboratory (University of Cambridge) or colleagues in other groups, as described in Tables S3. Genetic parts were amplified using primers listed in Table S2 and assembled into plasmid constructs following standard Golden Gate (GG) cloning according to the modular cloning (MoClo) system (Engler et al., 2014; Weber et al., 2011). If necessary, parts were domesticated by removing BpiI and BsaI sites using PCR‐based mutagenesis. All parts were verified by Sanger sequencing (Azenta, Takeley, UK).

### Detection of fluorescent reporter proteins

*P. tricornutum* strains transformed with different fluorescent reporter proteins were grown in 200 μL f/2 media with zeocin, and if required, in various B_12_ concentrations in 96‐well plates before measuring OD_730_, as a proxy for cell density, and fluorescence with a Clariostar plate reader (BMG Labtech, Ortenberg, Germany). Chlorophyll fluorescence was quantified by excitation at 440–449 nm and emission at 680–720 nm, and mVenus fluorescence was quantified by excitation at 515–510 nm and emission at 550–610 nm. mVenus fluorescence values were normalised to OD_730_ or chlorophyll fluorescence.

Transformants were imaged using a confocal laser scanning microscope (TCS SP5 or SP8; Leica Microsystems, Wetzlar, Germany). Images were acquired with excitation at 515 nm and emission 535–565 nm for mVenus or 650–720 nm for chlorophyll. The Leica LAS software was used to take pictures with a line average of 4. The smart gain was adjusted, as indicated in the figures, between 50 and 100% depending on the level of fluorescence. The pinhole was set to 2 AU for all imaging experiments.

### Metabolite analysis

*P. tricornutum* lines transformed with the casbene synthase expression cassette were assessed for casbene production by culturing individual lines either in 20 ml cultures in Nunc flasks (Thermo Fisher Scientific) or 2 ml cultures in 24‐well plates (Starlab) in f/2 media with various B_12_ concentrations. After 2 days, a 10% (v/v) dodecane overlay was added to capture the casbene. Samples of dodecane were removed at defined intervals, clarified by centrifugation, and mixed 1:1 with hexane containing 10 μg × ml^−1^ of the internal standard α‐caryophyllene. One microlitre of this mixture was injected and analysed by GC–MS (Thermo Fisher Scientific) using the method previously described (Lauersen et al., 2018). Casbene was quantified using selected ion spectra with a mass range of 119–121 (m/z). Total chlorophyll and carotenoid concentrations were determined after extraction of the cell pellet with dimethylformamide (DMF; Fisher, Loughborough, UK) using the equations from Jeffrey and Humphrey (1975) and from Wellburn (1994), respectively.

### Data sources and DNA sequence motif search

The gene, transcript and protein models, and functional annotations for *P*. tricornutum (ASM15095v2) were downloaded from Ensembl Protists. Transcriptome and protein data sets under B_12_ limitation conditions were obtained from Bertrand et al. (2012). For the motif search, the 500 bp upstream of the start codon of the *THIC* gene for diatom species *P. tricornutum, F. cylindrus, P. multistrata, T. pseudonana*, *C. cryptica* and *T. oceanica* were retrieved from Ensembl, JGI's Phycocosm or NCBI (gene ids can be found in Table S4), and used as a training set for the MEME algorithm (version 5.1.0, Bailey et al., 2009) with ‘Zero or One Occurrence per Sequence’ and leaving other parameters as default. From the hit with the lowest e‐value (6.7e+002) a 14 bp motif was generated as 5′‐GAAKYACGTKCWKC‐3′ (where K stands for G or T, Y for C or T, and W for A or T following the standard IUPAC nomenclature). To validate the motif, we chose the upstream region of (1) a set of genes known to be downregulated by B_12_ (*PtMETE1, FkMETE, PtCBA1, PtSHMT1, Tp42612 and Tp22483*) and (2) a selection of *P. tricornutum* genes for metabolic enzyme previously shown to be unaffected by B_12_ (Bertrand et al., 2012), *PtUBQ*, *PtH4*, *PtGGPPS*, *PtPSY*, *PtPDX1*, *PtUMPS*, *PtPDX2*, and *PtMETH* (Table S4). These were used as inputs for the MEME algorithm with the ‘Any Number of Repetition’ option and restricting the search to 25 bp long motifs. All 14 identified motifs (10 genes with single hits, one gene with four hits) were subsequently used to generate a consensus sequence using the standard settings (Weblogo3, https://weblogo.threeplusone.com, Crooks et al., 2004).

### Statistical analysis

Statistical analysis was carried out using an ANOVA test followed by Tukey's post‐hoc testing. The drc package with Weibull parameters was used to quantify the effective dose (ED) (Ritz et al., 2015).

## AUTHOR CONTRIBUTIONS

PRH, KG, and AGS designed the research; PRH, KG, ACF, MLP, and SN carried out experiments and analysed data; MLP and AH carried out the bioinformatics analysis; KG, GIMO, and PM supervised aspects of the project and analysed data. PRH, KG, and AGS wrote the article with input from all authors. AGS conceived the project, obtained the funding, and supervised the project. AGS agrees to serve as the author responsible for contact and ensures communication.

## CONFLICT OF INTEREST STATEMENT

The authors declare no commercial or financial conflict of interest.

## Supporting information


**Figure S1.** Genomic context of *PtMETE* and *PtCBA1* genes and neighbouring genes. (a) *PtMETE* gene showing promoter (P), 5′UTR (5), exons, intron, 3′UTR (3), and terminator. (b) *PtCBA1* gene. Arrows indicate the orientation of the genes. Numbers on top specify distances from the ATG start codon.

**Figure S2.** Confocal microscope images of transformed *P. tricornutum cells*. Transformants of P_*METE*_, P_*CBA1*_, and P_*FCPA*_ were analysed by confocal microscopy. Shown are the different channels for brightfield, mVenus fluorescence, and chlorophyll fluorescence (from left to right). Cells were grown without or with B_12_ (1 μg  L^−1^) and imaged after 4 days. To detect mVenus fluorescence, the gain was adjusted for the different lines, with the values shown underneath. Images were taken with excitation at 515 nm and emission for mVenus at 535–565 nm and emission for chlorophyll at 650–720 nm.

**Figure S3.** Influence of frequent subculturing on transgene expression. A representative line of P_*METE*_ (#C3) was grown in 96‐well plates in f/2 media in the absence of B_12_ (black symbols) or 1 μg L^−1^ B_12_ (red symbols). Media was inoculated with cells at the beginning of the experiment (left‐hand column) or subcultured in fresh f/2 media at a 2/3 dilution every 3 days (right‐column, arrowed). mVenus fluorescence, chlorophyll (Chl) fluorescence, and OD_730_ were recorded over time and plotted. mVenus fluorescence normalised to Chl is shown in the bottom panel. Error bars represent the standard deviation of three biological replicates. AU, arbitrary units.

**Figure S4.** Transgene expression in P_*AP1*_ is tuneable. Four independent P_*AP1*_ lines (#A3, #B2, #C3, #G1) which exhibited the highest fluorescence signal under normal f/2 conditions (36 mm phosphate), were transferred to media containing no phosphate (blue) or media containing five times the normal phosphate concentration (grey, 5xP, 180 mm phosphate). mVenus fluorescent signal, Chl fluorescence, and OD_730_ were recorded over time. Error bars represent the standard deviation of three biological replicates.

**Figure S5.** Modulation of *P*_*METE*_‐mVenus expression by B_12_. A *P. tricornutum* P_METE_ line was grown in 1 μg L^−1^ B_12_ for 7 days, then pelleted, washed, and resuspended in media without B_12_ or with different B_12_ concentrations (10, 50, 100 and 250 ng L^−1^). mVenus and Chl fluorescence and OD_730_ were recorded every 6 h. (*n* = 4, error bars represent SD).

**Figure S6.** Steady‐state transcript levels in P_*METE*_ and P_*METE*_T_*METE*_ lines. *P. tricornutum* cells transformed either with the P_*METE*_ or P_*METE*_T_*METE*_ construct were grown in f/2 without supplementation (−B_12_) or with 1 μg L^−1^ B_12_ (+B_12_). After 24 h, RT‐qPCR was performed with primers for the endogenous gene of the B_12_‐independent isoform methionine synthase (*METE*) and the transgene *mVenus*. The ΔΔCq values are shown normalised to the housekeeping gene histone H4. Values represent the mean of 3 biological replicates where error bars represent the standard deviation.

**Figure S7.** Identifying 14 nt motif in promoter region of diatom B_12_‐regulated genes. (a) To investigate the presence of a common motif in B_12_‐regulated genes, the upstream regions (500 bp) of *THIC* genes (known to be downregulated by B_12_ supplementation; Llavero‐Pasquina et al., 2022) were taken from six diatoms and used as training sequences with the MEME algorithm. A region of 14 nt (highlighted in green) was found in all six genes. It was then used to screen other B_12_‐regulated genes from diatoms and was found in *P. tricornutum METE*, *CBA1*, *SHMT1*, *Fragilariopsis kerguelensis* (*Fk*) *METE*, and *Thalassiosira pseudonana Tp2612* (highlighted in cyan). (b) Schematic showing the position of the motif(s) in the training and test sequences. The *PtMETE* gene has four copies, while all other genes have only one. *Cc*, *Cyclotella cryptica*; *Fc*, *Fragilariopsis cylindrus*; *Pm*, *Pseudonitzschia multiseries*; *To, Thalassiosira oceanica*; (c) Sequence logo for the conserved motif, built with WebLogo3 using the sequences of diatom promoters from 11 genes downregulated by B_12_ supplementation (Table S4).

**Figure S8.** Identification of casbene producing *P. tricornutum* lines. P_*METE*_ *CS* lines were screened for casbene production after 5 days of incubation (3 days after addition of dodecane overlay). Total casbene was calculated from the area under the peak corresponding to the casbene ion fragmentation pattern on GC–MS spectra. Error bars represent the standard deviation of three biological replicates.

**Figure S9.** Influence on carotenoids and chlorophyll in *P. tricornutum* cells producing casbene. Cultures of P_*METE*_ *CS* line #F05 were grown without B_12_ (black) and with various B_12_ concentrations (50–1000 ng L^−1^). A dodecane overlay was added 2 days post‐inoculation, and at day 11 post‐inoculation (a) carotenoid levels and (b) chlorophyll levels of the cultures were analysed in DMF‐extracts by UV–Vis spectrometry. Error bars represent the standard deviation of three biological replicates.

**Table S1.** Summary of selected transcriptomic studies of *P. tricornutum* under nutrient‐stress conditions. Nutrient stress, analytic method, and time point at which samples were analysed after applying the nutrient stress are summarised. Each study calculated the percentage of genes or transcripts with a differential gene expression (DGE) after applying the nutrient stress.
**Table S2.** Oligonucleotides used in this study. Forward (fw) and reverse (rv) primers used in this study. Restriction sites used for cloning are indicated in bold. Underlined nucleotides are specific 4 nt overhangs used for Golden Gate‐based cloning.
**Table S3.** Plasmids used in this study. The majority of plasmids were assembled following a Golden Gate cloning strategy and using previously developed parts (pCM0, Crozet et al. 2018) and backbone plasmids (pICH, Engler et al., 2014).
**Table S4.** Sequence information for motif search. Summary of motifs found in diatom THIC genes and other genes downregulated by cobalamin supplementation. The position denotes the distance between the 3′ end of the motif and the start codon of the gene. The genes whose promoter regions were used to find the motif with the MEME software are labelled as ‘Training’; those that have been experimentally confirmed to be downregulated by cobalamin supplementation and have not been used in the MEME search are labelled ‘Test’. The 5′ and 3′ flanking sequences for the motif are included. PtMETE1‐4 correspond to the blue boxes in Figure 6. As negative controls, eight genes that are not influenced by cobalamin supplementation have also been searched for the motif. The gene ID, assembly, sequence source, and a brief description of protein function are listed.

**Data S1.** Supporting Information.

## Data Availability

Data associated with this manuscript are either included in the Supplementary Information or available in the data repository of the University of Cambridge, UK (data.cam.ac.uk/repository).
