# Supplementary figures and images for: A new tool for engineering Phaeodactylum tricornutum: the METE promoter drives both high expression and B12 ‐tuneable regulation of transgenes
Source: Plant J. 2025 Oct 21;124(2):e70210. doi: 10.1111/tpj.70210 (PMC12538271; doi:10.1111/tpj.70210)

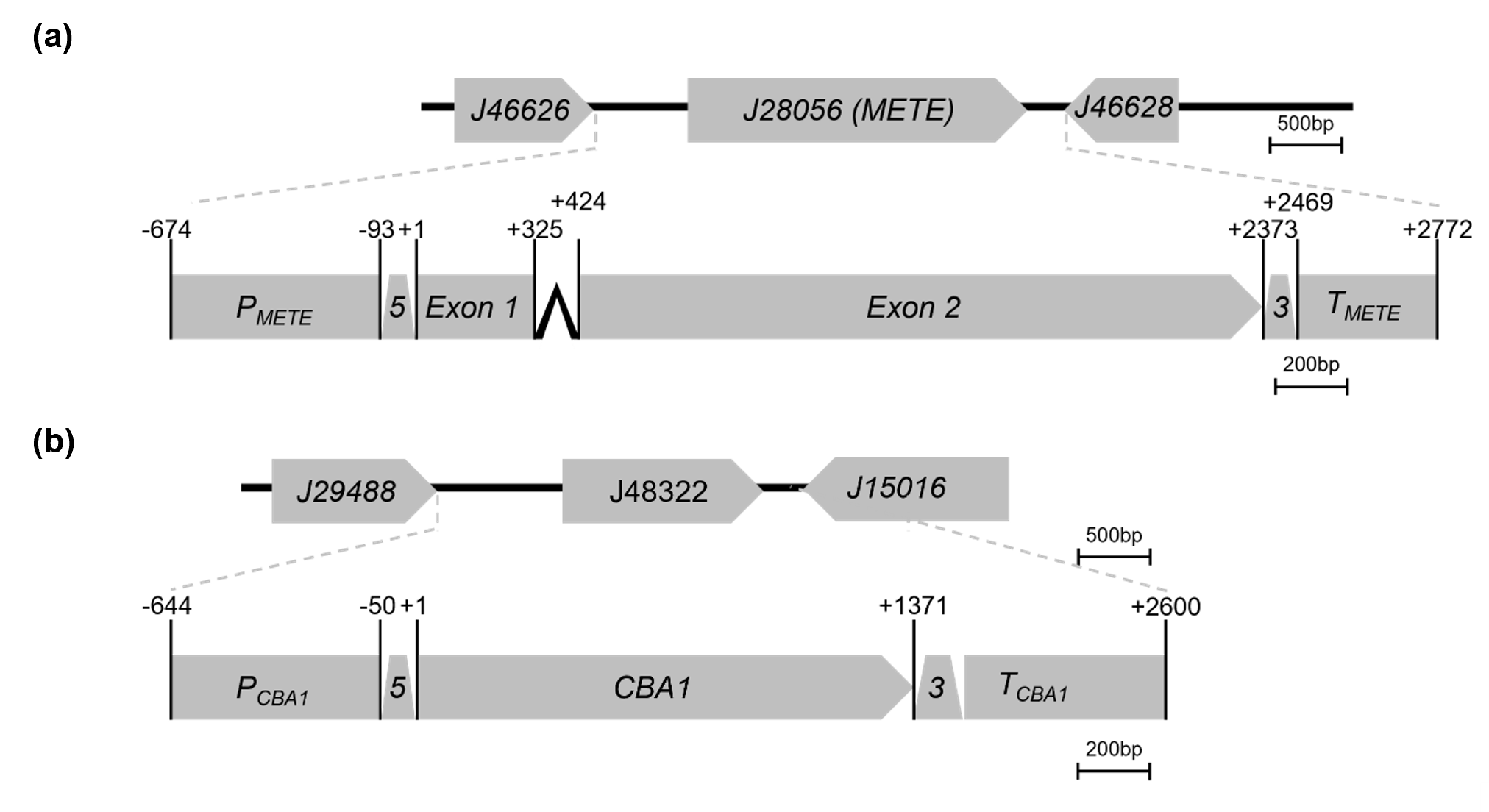

Supplement: Supplementary file 1 — Figure S1. Genomic context of PtMETE and PtCBA1 genes and neighbouring genes. (a) PtMETE gene showing promoter (P), 5′UTR (5), exons, intron, 3′UTR (3), and terminator. (b) PtCBA1 gene. Arrows indicate the orientation of the genes. Numbers on top specify distances from the ATG start codon. [file TPJ-124-0-s002.png]

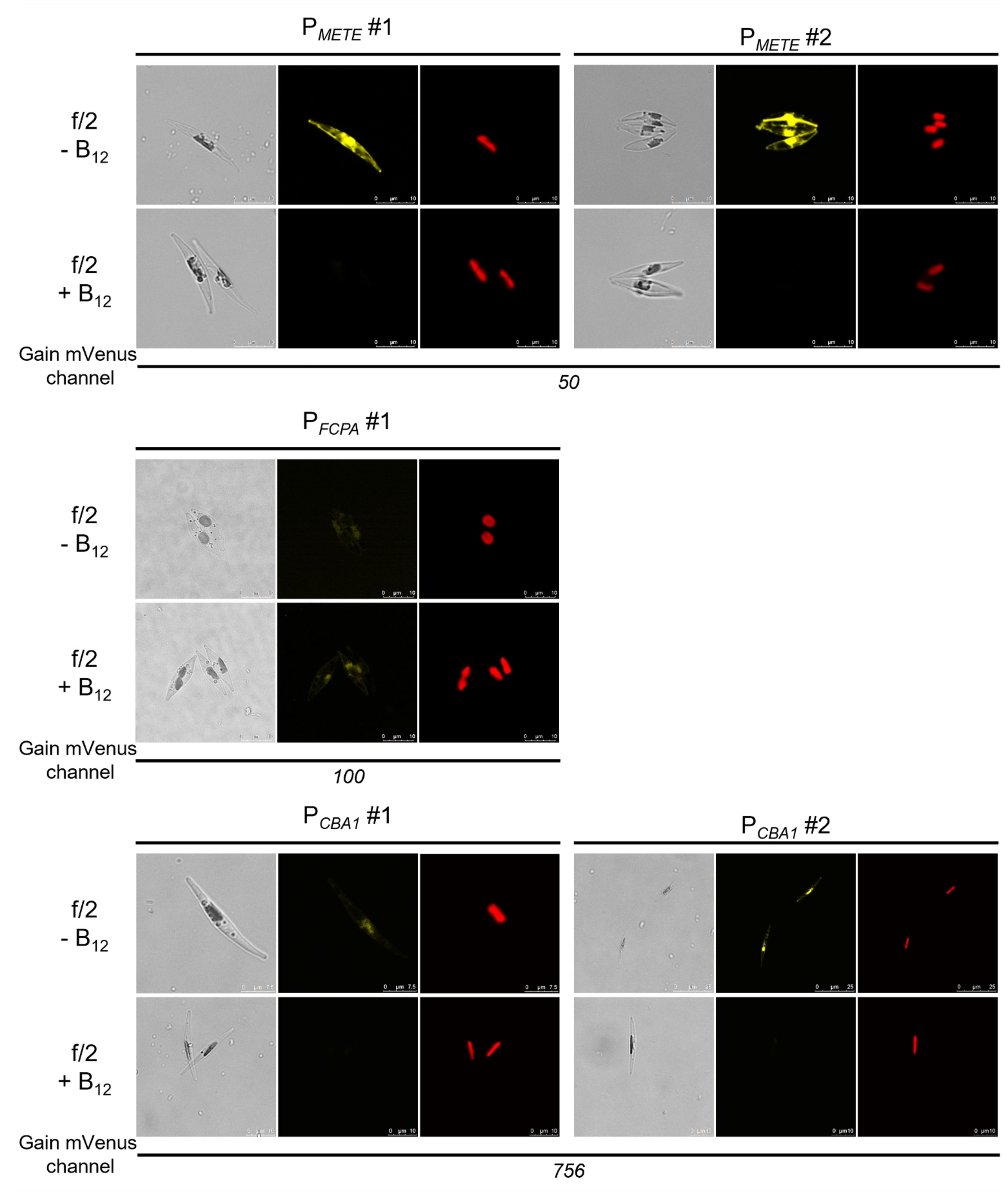

Supplement: Supplementary file 2 — Figure S2. Confocal microscope images of transformed P. tricornutum cells. Transformants of P METE , P CBA1 , and P FCPA were analysed by confocal microscopy. Shown are the different channels for brightfield, mVenus fluorescence, and chlorophyll fluorescence (from left to right). Cells were grown without or with B12 (1 μg L−1) and imaged after 4 days. To detect mVenus fluorescence, the gain was adjusted for the different lines, with the values shown underneath. Images were taken with excitation at 515 nm and emission for mVenus at 535–565 nm and emission for chlorophyll at 650–720 nm. [file TPJ-124-0-s006.png]

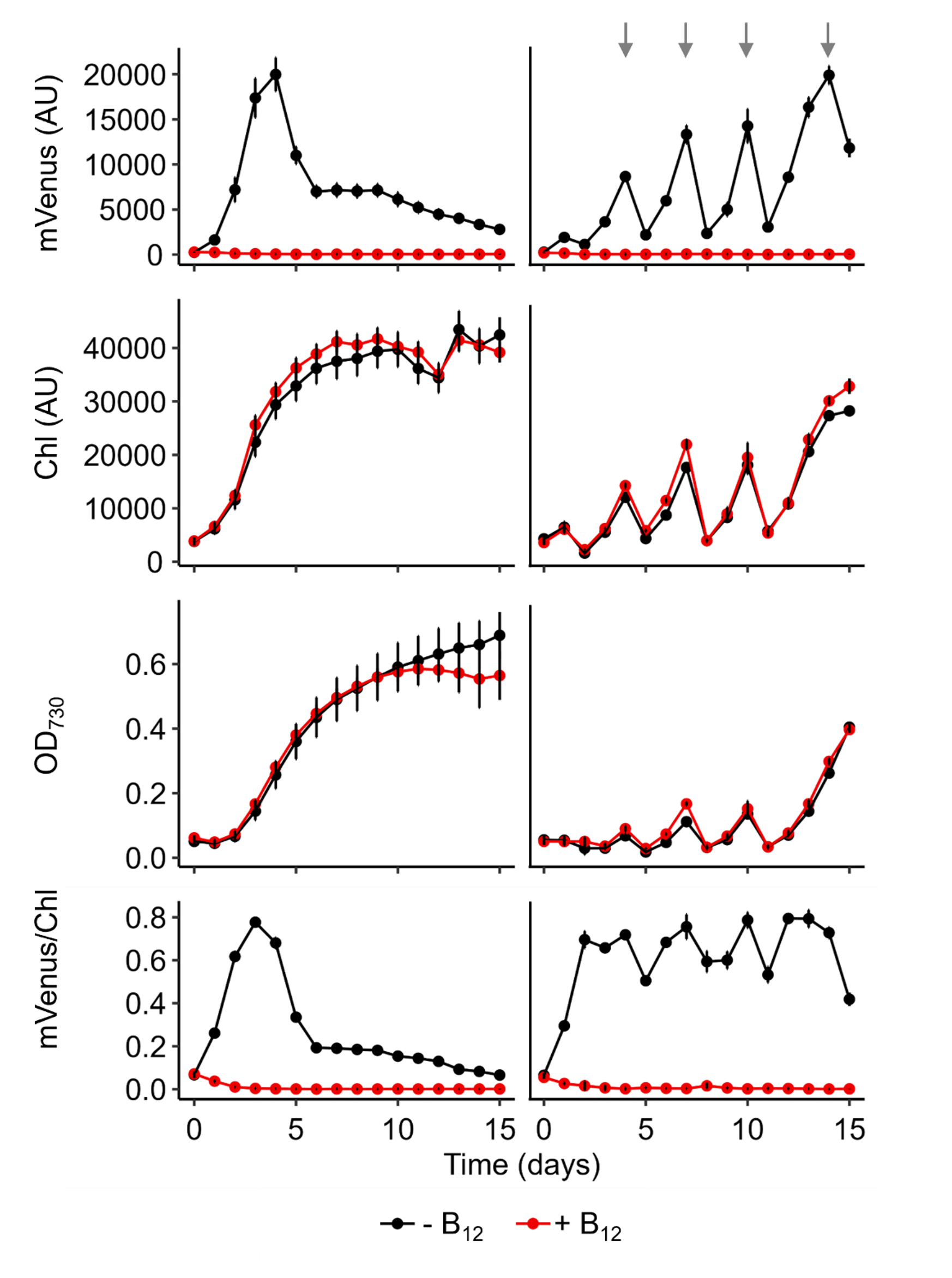

Supplement: Supplementary file 3 — Figure S3. Influence of frequent subculturing on transgene expression. A representative line of P METE (#C3) was grown in 96‐well plates in f/2 media in the absence of B12 (black symbols) or 1 μg L−1 B12 (red symbols). Media was inoculated with cells at the beginning of the experiment (left‐hand column) or subcultured in fresh f/2 media at a 2/3 dilution every 3 days (right‐column, arrowed). mVenus fluorescence, chlorophyll (Chl) fluorescence, and OD730 were recorded over time and plotted. mVenus fluorescence normalised to Chl is shown in the bottom panel. Error bars represent the standard deviation of three biological replicates. AU, arbitrary units. [file TPJ-124-0-s008.png]

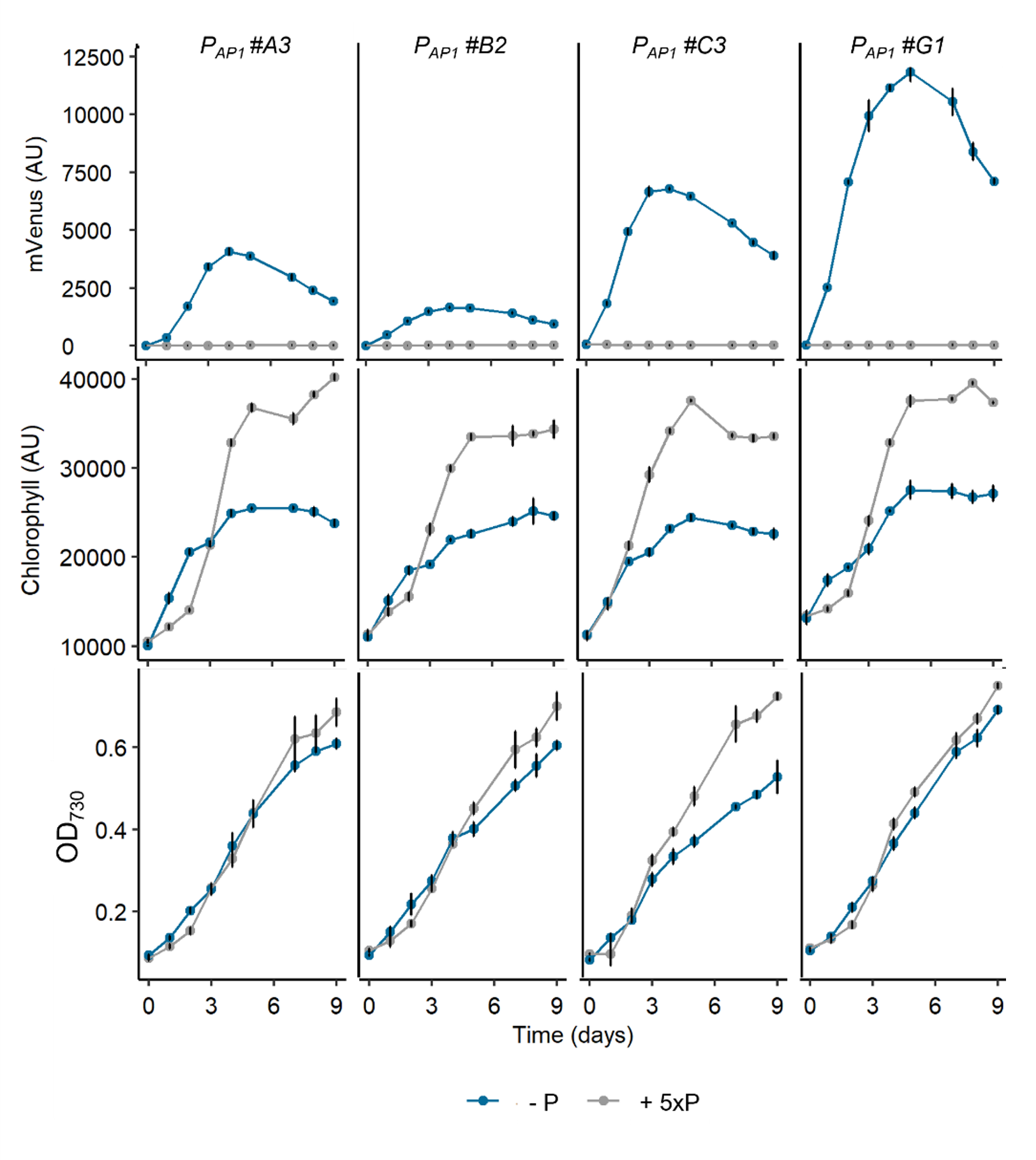

Supplement: Supplementary file 4 — Figure S4. Transgene expression in P AP1 is tuneable. Four independent P AP1 lines (#A3, #B2, #C3, #G1) which exhibited the highest fluorescence signal under normal f/2 conditions (36 mm phosphate), were transferred to media containing no phosphate (blue) or media containing five times the normal phosphate concentration (grey, 5xP, 180 mm phosphate). mVenus fluorescent signal, Chl fluorescence, and OD730 were recorded over time. Error bars represent the standard deviation of three biological replicates. [file TPJ-124-0-s004.png]

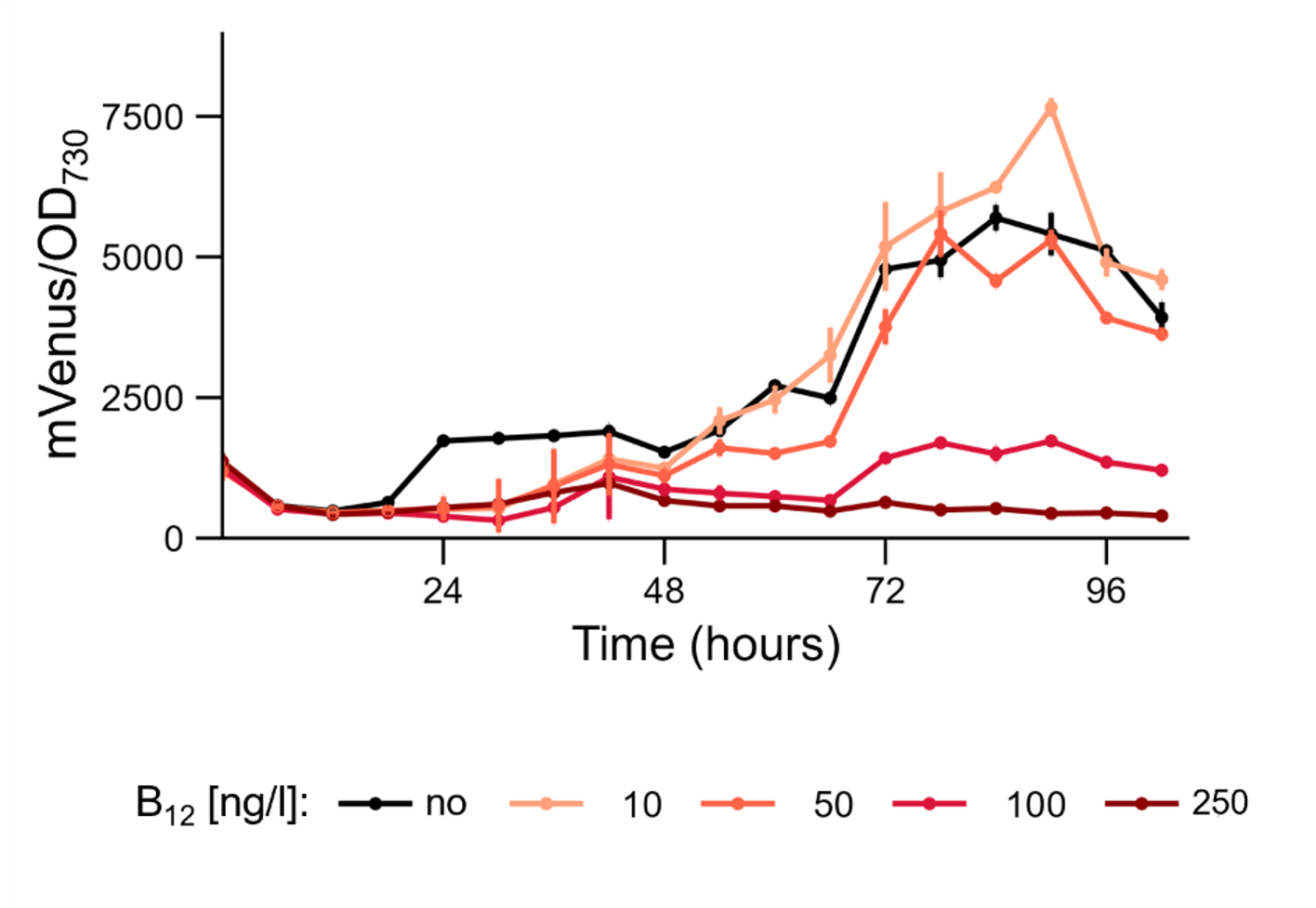

Supplement: Supplementary file 5 — Figure S5. Modulation of P METE ‐mVenus expression by B12. A P. tricornutum PMETE line was grown in 1 μg L−1 B12 for 7 days, then pelleted, washed, and resuspended in media without B12 or with different B12 concentrations (10, 50, 100 and 250 ng L−1). mVenus and Chl fluorescence and OD730 were recorded every 6 h. (n = 4, error bars represent SD). [file TPJ-124-0-s010.png]

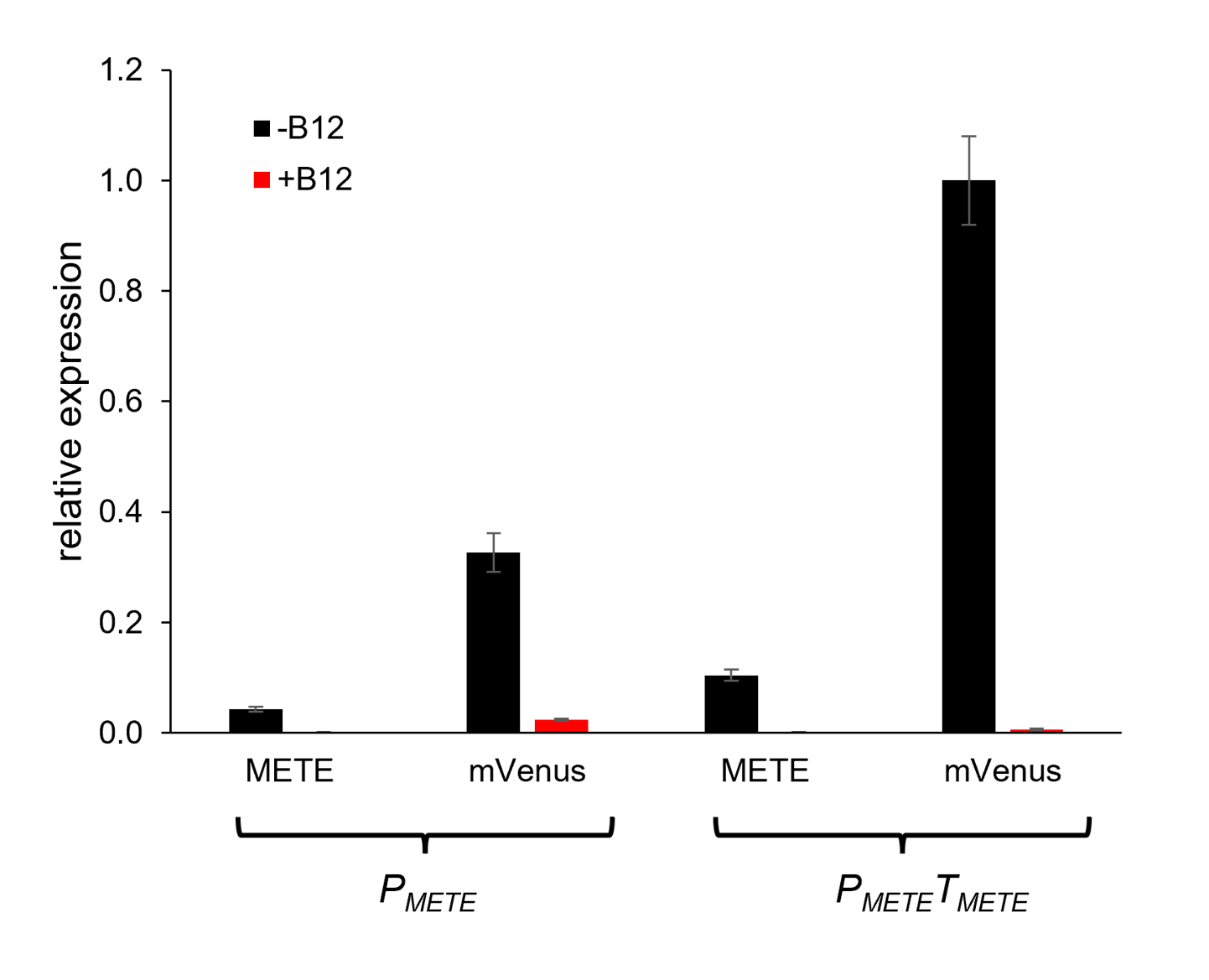

Supplement: Supplementary file 6 — Figure S6. Steady‐state transcript levels in P METE and P METE T METE lines. P. tricornutum cells transformed either with the P METE or P METE T METE construct were grown in f/2 without supplementation (−B12) or with 1 μg L−1 B12 (+B12). After 24 h, RT‐qPCR was performed with primers for the endogenous gene of the B12‐independent isoform methionine synthase (METE) and the transgene mVenus. The ΔΔCq values are shown normalised to the housekeeping gene histone H4. Values represent the mean of 3 biological replicates where error bars represent the standard deviation. [file TPJ-124-0-s011.png]

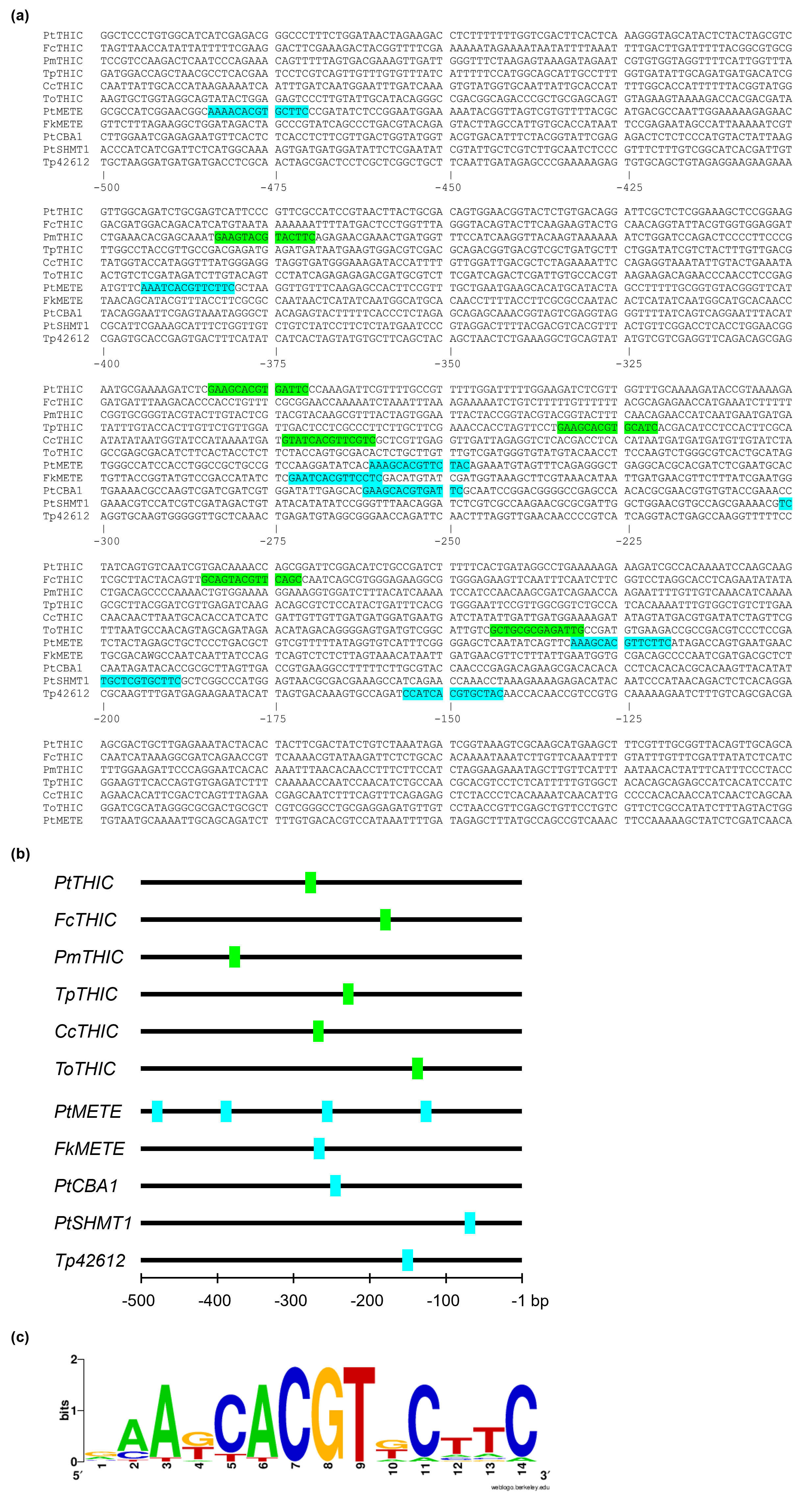

Supplement: Supplementary file 7 — Figure S7. Identifying 14 nt motif in promoter region of diatom B12‐regulated genes. (a) To investigate the presence of a common motif in B12‐regulated genes, the upstream regions (500 bp) of THIC genes (known to be downregulated by B12 supplementation; Llavero‐Pasquina et al., 2022) were taken from six diatoms and used as training sequences with the MEME algorithm. A region of 14 nt (highlighted in green) was found in all six genes. It was then used to screen other B12‐regulated genes from diatoms and was found in P. tricornutum METE, CBA1, SHMT1, Fragilariopsis kerguelensis (Fk) METE, and Thalassiosira pseudonana Tp2612 (highlighted in cyan). (b) Schematic showing the position of the motif(s) in the training and test sequences. The PtMETE gene has four copies, while all other genes have only one. Cc, Cyclotella cryptica; Fc, Fragilariopsis cylindrus; Pm, Pseudonitzschia multiseries; To, Thalassiosira oceanica; (c) Sequence logo for the conserved motif, built with WebLogo3 using the sequences of diatom promoters from 11 genes downregulated by B12 supplementation (Table S4). [file TPJ-124-0-s003.png]

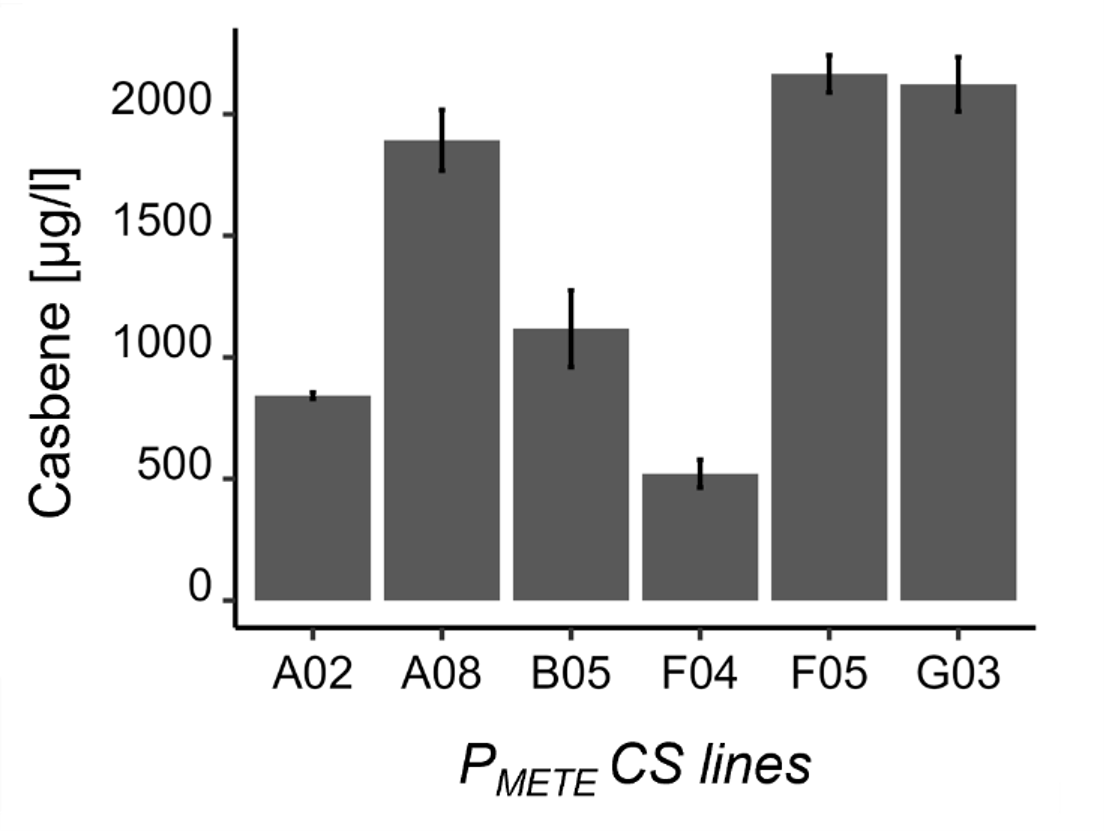

Supplement: Supplementary file 8 — Figure S8. Identification of casbene producing P. tricornutum lines. P METE CS lines were screened for casbene production after 5 days of incubation (3 days after addition of dodecane overlay). Total casbene was calculated from the area under the peak corresponding to the casbene ion fragmentation pattern on GC–MS spectra. Error bars represent the standard deviation of three biological replicates. [file TPJ-124-0-s005.png]

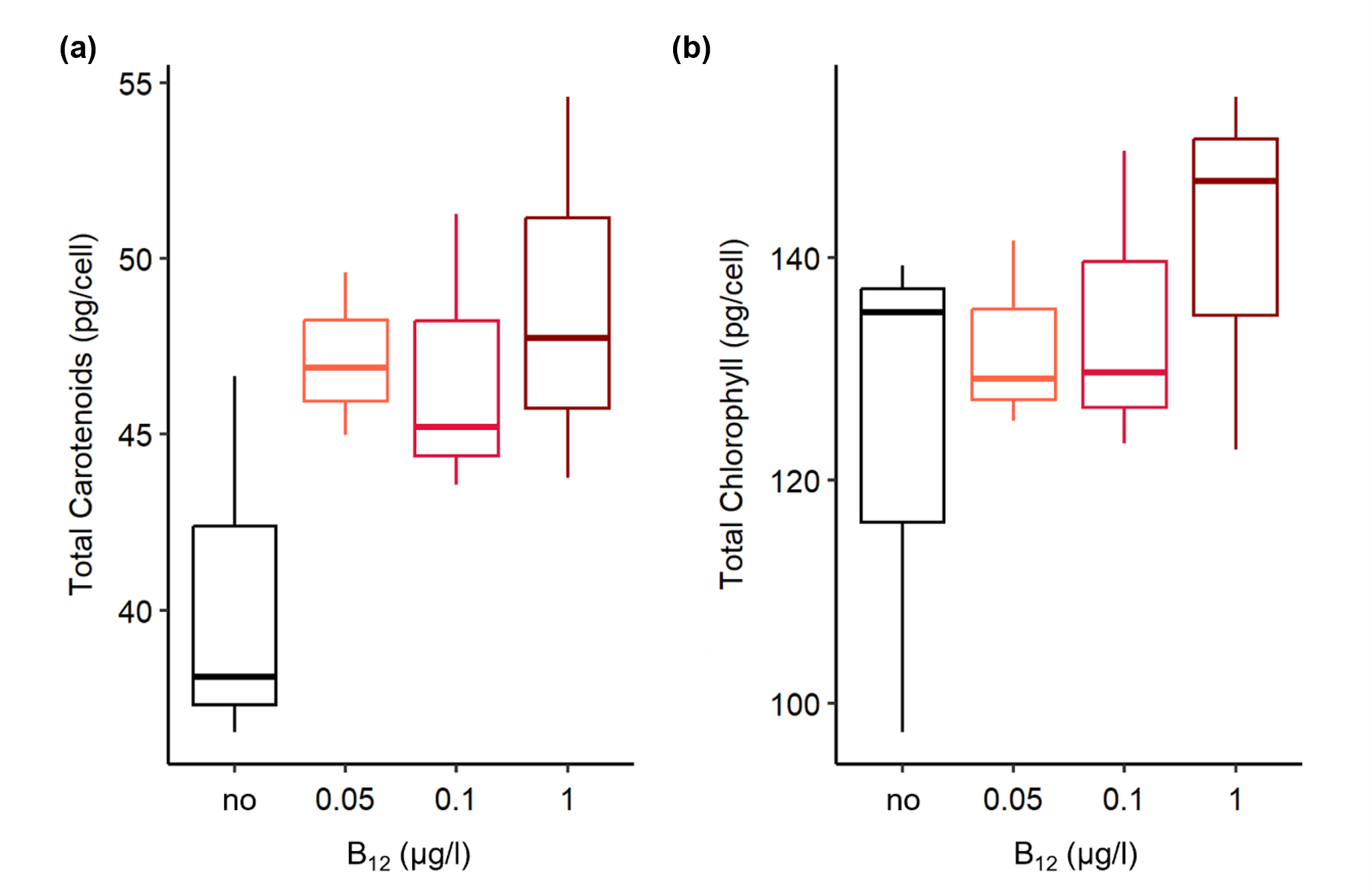

Supplement: Supplementary file 9 — Figure S9. Influence on carotenoids and chlorophyll in P. tricornutum cells producing casbene. Cultures of P METE CS line #F05 were grown without B12 (black) and with various B12 concentrations (50–1000 ng L−1). A dodecane overlay was added 2 days post‐inoculation, and at day 11 post‐inoculation (a) carotenoid levels and (b) chlorophyll levels of the cultures were analysed in DMF‐extracts by UV–Vis spectrometry. Error bars represent the standard deviation of three biological replicates. [file TPJ-124-0-s001.png]
